# Risk of Cardiovascular Disease Hospitalization After Common Psychiatric Disorders: Analyses of Disease Susceptibility and Progression Trajectory in the UK Biobank

**DOI:** 10.1007/s43657-023-00134-w

**Published:** 2024-07-08

**Authors:** Xin Han, Yu Zeng, Yanan Shang, Yao Hu, Can Hou, Huazhen Yang, Wenwen Chen, Zhiye Ying, Yajing Sun, Yuanyuan Qu, Junren Wang, Wei Zhang, Fang Fang, Unnur Valdimarsdóttir, Huan Song

**Affiliations:** 1 Mental Health Center and West China Biomedical Big Data Center, West China Hospital, Sichuan University, Guo Xue Lane 37, Chengdu, 610000 China; 2 Med-X Center for Informatics, Sichuan University, Chengdu, 610000 China; 3 Division of Nephrology, Kidney Research Institute, State Key Laboratory of Biotherapy and Cancer Center, West China Hospital, Sichuan University, Chengdu, 610000 China; 4 Mental Health Center, West China Hospital of Sichuan University, Chengdu, 610000 China; 5 Institute of Environmental Medicine, Karolinska Institutet, 17177 Stockholm, Sweden; 6 Center of Public Health Sciences, Faculty of Medicine, University of Iceland, 102 Reykjavík, Iceland; 7 Department of Medical Epidemiology and Biostatistics, Karolinska Institutet, 17177 Stockholm, Sweden; 8 Department of Epidemiology, Harvard T H Chan School of Public Health, Boston, MA 02115 USA

**Keywords:** Psychiatric disorders, Cardiovascular disease, Disease susceptibility, Disease trajectory

## Abstract

Whether associations between psychiatric disorders and hospitalization for cardiovascular diseases (CVDs) can be modified by disease susceptibility and the temporal pattern of these associated CVDs remain unknown. In our study, we conducted a matched cohort study of the UK Biobank including 44,430 patients with common psychiatric disorders (anxiety, depression, and stress-related disorders) between 1997 and 2019, together with 222,150 sex-, Townsend deprivation index-, and birth year- individually matched unexposed individuals. The hazard ratios (HRs) for CVD hospitalization associated with a prior psychiatric disorder were derived from Cox models, adjusted for multiple confounders. We then stratified the analyses by self-reported family history of CVD and CVD polygenic risk score (PRS) calculated based on summary statistics of independent genome-wide association studies. We further conducted disease trajectory analysis and visualized the temporal pattern of CVDs after common psychiatric disorders. During a mean follow-up of 12.28 years, we observed an elevated risk of CVD hospitalization among patients with psychiatric disorders, compared with matched unexposed individuals (hazard ratios [HRs] = 1.20, 95% confidence interval [CI]: 1.18–1.23), especially during the first six months of follow-up (1.72 [1.55–1.91]). The stratification analyses by family history of CVD and by CVD PRS obtained similar estimates between subgroups with different susceptibilities to CVD. We conducted trajectory analysis to visualize the temporal pattern of CVDs after common psychiatric disorders, identifying primary hypertension, acute myocardial infarction, and stroke as three main intermediate steps leading to further increased risk of other CVDs. In conclusion, the association between common psychiatric disorders and subsequent CVD hospitalization is not modified by predisposition to CVD. Hypertension, acute myocardial infarction, and stroke are three initial CVDs linking psychiatric disorders to other CVD sequelae, highlighting a need of timely intervention on these targets to prevent further CVD sequelae among all individuals with common psychiatric disorders.

**Supplementary Information:**

The online version contains supplementary material available at 10.1007/s43657-023-00134-w.

## Introduction

Both psychiatric disorders and cardiovascular diseases (CVDs) are leading causes of disability, morbidity, and mortality worldwide according to the World Health Organization (WHO) (Whiteford et al. 2013; DALYs GBD et al. 2015). In recent decades, accumulating evidence has indicated a link between psychiatric disorders and CVD (Kollia et al. 2017; Song et al. 2019; Momen et al. 2020; Han et al. 2021). Specifically, elevated risks of multiple CVDs, including ischemic heart disease (Janszky et al. 2010; Wium-Andersen et al. 2019), hypertension (Stein et al. 2014), heart failure (Song et al. 2019), atherosclerosis (Yu et al. 2010), and stroke (Swain et al. 2015; Momen et al. 2020), have been reported among patients with psychiatric disorders. Potential mechanisms linking psychiatric disorders and cardiovascular abnormalities are multifactorial, including chronic inflammation (e.g., representing as elevated serum levels of C-reactive protein and interleukin-6 (Khandaker et al. 2020)), alterations in the hypothalamic–pituitary–adrenal (HPA) axis (Zorn et al. 2017), abnormal immune response (e.g., changes of serum complement C3 and component 1qC levels Lee et al. 2015; Tao et al. 2020)), and unhealthy lifestyle (e.g., smoking) (Weinberger et al. 2017).

Recent genome-wide association studies (GWAS) have suggested a genetic predisposition to different CVDs, such as coronary artery disease (Consortium CAD et al. 2013; Nikpay et al. 2015) and stroke (SiGN and ISGC 2016), identifying multiple significantly associated single-nucleotide polymorphisms (SNPs), with a reported heritability ranging from 10.6 to 13.3%. Likewise, familial factors, as a proxy for the joint impact of genetic background and shared environmental and lifestyle factors, have been shown to play an important role in the development of CVD (Forsdahl 1979; Iannotti et al. 2000). However, data are scarce regarding whether predisposition to CVD can modify the association between psychiatric disorders and subsequent CVD. Findings from a Danish twin study did not support a role of shared familial factors in the association of depression with CVD (Wium-Andersen et al. 2020). In our previous study, however, we noted attenuated risk increase of CVD among patients with stress-related disorders when comparing them with their full siblings, relative to comparison with age- and sex-matched unexposed individuals from the general population (Song et al. 2019). These inconsistent results call for further investigations of this question, ideally with direct assessment of disease susceptibility to CVD such as individual-level genotype data.

Moreover, since different CVDs have shared etiologies (The Emerging Risk Factors Collaboration 2015; Laurent and Boutouyrie 2015), it is possible that some specific CVDs are more directly affected by a prior psychiatric disorder, while other downstream CVDs emerge primarily due to inherent pathogenetic connections between CVDs. We therefore applied disease trajectory analysis to map out the temporal progression network of CVDs after psychiatric disorders (Siggaard et al. 2020; Han et al. 2021). The results of such analysis can be informative in terms of identifying key CVDs that could be potential targets for developing preventive measures, with the goal of effectively reducing the risk of subsequent CVDs among patients with psychiatric disorders.

In the present study, taking advantage of enriched phenotypic information, almost complete follow-up, and individual-level genotyping data in the UK Biobank, we aimed to clarify whether disease susceptibility to CVD (i.e., indicated by genetic predisposition and familial history) modifies the association of common psychiatric disorders with subsequent risk of hospitalization for CVDs. Furthermore, we used trajectory analysis to identify the key CVDs directly associated with a prior diagnosis of common psychiatric disorders.

## Materials and Methods

### Study Design

The UK Biobank is a community-based cohort study which enrolled half a million participants aged 40 to 69 years at recruitment between 2006 and 2010 across England, Scotland, and Wales. Written informed consent was obtained from all participants by the UK Biobank. The UK Biobank has full ethical approval from the National Research Ethics Service (NO. 16/NW/0274). Baseline questionnaires and biological samples were collected at recruitment with detailed descriptions elsewhere (Sudlow et al. 2015). Health-related outcomes were obtained by linkages to a range of health records (Sudlow et al. 2015). UK Biobank inpatient hospital data were mapped from the Hospital Episode Statistics database, the Scottish Morbidity Record, and the Patient Episode Database, covering 89% of UK Biobank participants since 1997. The primary care data were derived from various general practitioner computer system suppliers, providing information on primary care visits for 45% of participants since 1985 (Sudlow et al. 2015).

**Fig. 1:**
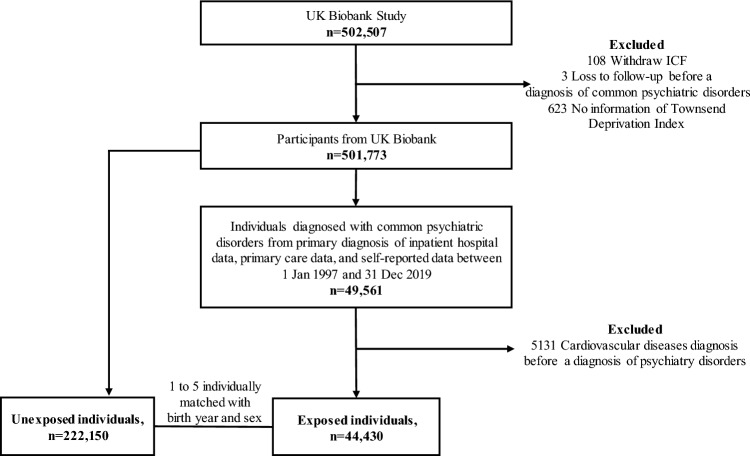
Flowchart of the participants' selection. This figure shows the study design, and the inclusion and exclusion process of study population selection

Based on these data, we conducted a matched cohort study. After the exclusion of 108 individuals who withdrew their informed consent forms, three individuals who lost to follow-up before a diagnosis of common psychiatric disorders, and 623 individuals without information on the Townsend deprivation index (TDI), we identified an exposed cohort of 49,561 participants who received their first diagnosis of anxiety, depression, or stress-related disorders between 1 January 1997 and 31 December 2019 (Fig. 1). The TDI score was assigned to each individual corresponding to the located postcode, with a higher TDI score indicating higher deprivation (Townsend et al. 1988). Individuals with those common psychiatric disorders were included in the exposed cohort from the date of their diagnosis (i.e., the index date). We then excluded individuals with a history of any CVD before the index date (*n* = 5131), leaving 44,430 exposed individuals in the final analysis. For each exposed individual (i.e., the index patient), we randomly selected five unexposed individuals per index patient, individually matched by sex, TDI (transformed to an ordinal variable by quartile), and birth year (± two years), from individuals who were free of those common psychiatric disorders and CVD at the diagnosis date of the index patient.

### Follow-Up

For all participants, follow-up was from the index date until the diagnosis of any or specific CVDs, death, loss to follow-up, or the end of follow-up (31 December 2019), whichever occurred first. The follow-up for matched unexposed individuals was additionally censored at the time of their first diagnosis of studied psychiatric disorders, if any, during the follow-up.

### Ascertainment of Common Psychiatric Disorders

Common psychiatric disorders were defined as a diagnosis of anxiety, depression, or stress-related disorders, based on the primary diagnoses from the UK Biobank inpatient hospital, primary care, and self-reported data. Table S1 lists details of diagnosis codes, including the ninth-to-tenth versions of the International Classification of Diseases (ICD) codes for inpatient hospital and self-reported data, and version two and version three read codes (i.e., Read v2 and Read v3) for primary care data. We considered a diagnosis of other psychiatric disorders before the index date as a history of other psychiatric disorders. Inpatient mental health diagnosis from the Hospital Episode Statistics database was validated with a median accuracy of 73% against a gold-standard research diagnosis (Davis et al. 2018), while the positive predictive value (PPV) of Read codes for anxiety and depression from primary care data was 61%–76% against the five-item Mental Health Inventory (John et al. 2016).

### Ascertainment of CVD

With a specific focus on severe CVD consequences that require hospital care, we defined an incident CVD (any CVD, including six major subtypes: hypertensive diseases, ischemic heart diseases, embolism and thrombosis, arrhythmia/conduction disorders, heart failure, and cerebrovascular disease) as a corresponding diagnosis documented in the UK Biobank inpatient hospital data, according to ICD-9 and ICD-10 codes (Table S1). We further identified a group of acute cardiovascular events, referring to acute and severe cardiac consequences, including acute myocardial infarction, cardiac arrest, subarachnoid bleeding, intracerebral bleeding, and cerebral infarction (see Table S1). For disease trajectory analysis, we used the 3-digit ICD-10 codes for CVD identification based on the UK Biobank inpatient hospital data and combined CVDs with clinical or biological similarities (Yang et al. 2019; Han et al. 2021) (Table S2). The inpatient diagnosis of CVDs in the UK Biobank has been validated, showing satisfactory accuracy (i.e., PPV = 72% for coronary heart disease and > 90% for stroke (Woodfield et al. 2015; Kivimaki et al. 2017)).

### Disease Susceptibility of CVD

In the present study, we assessed the disease susceptibility to CVD in two ways. First, we defined family history of CVD by a self-reported history of heart diseases and stroke among first-degree relatives (i.e., father, mother, and siblings), obtained from baseline questionnaires (Field ID: 20,107, 20,110, and 20,111). Second, polygenic risk score (PRS) for six subtypes of CVDs were calculated for 418,247 individuals with eligible genotyping data (13,225,429 SNPs available after standard quality control (Marees et al. 2018) with details shown in Fig. S1) using LDPred2 (Prive et al. 2020), a method for PRS computation based on a combination of summary statistics and a matrix of correlations between genetic variants. The independent SNPs associated with the corresponding CVD subtype were derived from publicly available GWAS summary data (Table S3). In a validation step, the generated PRSs were shown to be significantly associated with an increased risk of its corresponding CVD phenotype in our dataset (e.g., for ischemic heart disease, odds ratio [95% confidence intervals (CIs)]: 1.56 [1.53–1.58], per standard deviation (SD) increase of PRS, see Table S3).

### Covariates

We retrieved information on educational levels, ethnicity, smoking status, TDI, and body mass index (BMI) through the baseline questionnaires. Based on the UK Biobank inpatient hospital data, we calculated the Charlson comorbidity index (CCI) on the index date for each individual, as a proxy of their baseline comorbidity level (Quan et al. 2005).

### Statistical Analysis

We used a stratified Cox model (Holt and Prentice 1974) to estimate hazard ratios (HRs) with 95% CIs of any CVD hospitalization, in relation to a previous diagnosis of common psychiatric disorders, using time since the index date as the underlying time scale. The models were stratified by matched identifiers (unique ID for each exposed patient and their individually matched unexposed individuals), and partially or fully adjusted for sex, birth year, TDI, educational levels, ethnicity, smoking status, BMI, history of other psychiatric disorders, family history of CVD, and CCI. As a high-risk time window for CVD has been indicated after the diagnosis of psychiatric disorders (Song et al. 2019), we first visualized the changes in HRs over time by stratifying the Cox models to different follow-up periods (≤ 3, 3–6, 6–12, 12–18, 18–24, 24–60, 60–120, 120–240, and > 240 month follow-up). As the HR during the first six months of the follow-up period was greater than that at later times (Fig. S2), we assessed the risk of CVD within and beyond the first six months (i.e., ≤ 6 or > 6 months of follow-up) separately in later analyses. In addition, we performed separate analyses for six main categories of CVDs and acute cardiovascular events, as well as for anxiety, depression, and stress-related disorders. Additionally, we detected the effect of severity, as well as onset age, of studied psychiatric disorders on the associations of interest through subgroup analyses by diagnosis sources (i.e., only self-reported/primary care diagnosis and inpatient diagnosis) and by age at diagnosis (i.e., < 47, 47–55, and > 55 years). The statistical significance of the difference between sub-grouped HRs was assessed by the Wald test.

To determine the impact of disease susceptibility to CVD on the association between common psychiatric disorders and CVD, we conducted stratified analyses by family history of CVD (yes or no, for any CVD) and by level of CVD PRS (high, moderate, or low by tertile distribution, for six subtypes of CVD).

The temporal progression of CVD subsequent to a diagnosis of psychiatric disorders was explored using disease trajectory analysis (Siggaard et al. 2020), which is an approach that has been successfully applied for detecting directional networks of comorbidities after a diagnosis of depression (Han et al. 2021) and breast cancer (Yang et al. 2019). In brief, this method includes three interrelated steps (see Fig. S3), with detailed descriptions in the Supplementary Methods. First, Cox regressions were performed to identify associations between 26 specific CVDs (see details ICD-10 codes in Table S2) and a prior diagnosis of psychiatric disorders. In this step, the *p* value for statistical significance was set to 0.05/number of analyses performed (Bonferroni corrections), to account for multiple testing. Among these significant CVDs (i.e., HR > 1 and *p* < Bonferroni corrected threshold) from the last step, we constructed all possible CVD1 and CVD2 pairs. We then used binomial tests to identify pairs of CVD events with a temporal order (i.e., CVD1 → CVD2, *p* < Bonferroni corrected threshold). For each CVD1 → CVD2 pair, we assessed the magnitude of the associations between CVD pairs through a nested case–control study design and conditional logistic regression (i.e., identifying CVD2 cases with CVD2-free individuals at the same time and contrasting their odds of having a prior CVD1 diagnosis, see Fig. S3). Only CVD pairs with confirmed associations (i.e., odds ratio [OR] > 1 and *p* < Bonferroni corrected threshold in the nested case–control analyses) were included in the construction of the trajectory network. The trajectory network of CVDs was formed by combining CVD pairs with overlapping CVD (e.g., disease pairs CVD1 → CVD2 and CVD2 → CVD3 with overlapping CVD2 were combined in the trajectory CVD1 → CVD2 → CVD3). In the trajectory network, each node represents a CVD, linked by lines with arrow (i.e., temporal direction of CVD pairs), and nodes (i.e., CVDs) located at the prior layers indicate that they were affected earlier by the studied psychiatric disorders.

To test the robustness of our results to the definition of CVD, we reassessed these associations by identifying CVD solely based on the primary diagnosis from UK Biobank inpatient hospital data. All statistical analyses were conducted by R (version 4.0.2), PLINK (version 1.9), Python (version 3.8), and Cytoscape (version 3.8.0).

## Results

In total, we identified 44,430 patients with studied common psychiatric disorders, together with 222,150 sex-, TDI-, and birth year-matched unexposed individuals (Fig. 1). Table 1 shows the basic characteristics of the study population. The mean age at index date was 51.14 years, and 65.18% of participants were females. Compared with matched unexposed individuals, patients with common psychiatric disorders were more likely to be smokers (either previous or current, 47.75% vs 41.76%), have a history of other psychiatric disorders (12.31% vs 3.79%), have lower educational level (41.22% vs 44.86% for university level), but higher BMI (27.40% vs 22.43% for more than 30 kg/M^2^).

**Table 1:** Baselines characteristics of the study cohort

	Total individuals (*n* = 266,580)	Individuals with psychiatry disorders (*n* = 44,430)	Matched individuals without psychiatry disorders^1^ (*n* = 222,150)
Follow-up time (years), mean (SD)	12.28 (5.78)	12.25 (5.79)	12.29 (5.78)
Age at index date, mean (SD)	51.14 (8.90)	51.10 (8.94)	51.15 (8.89)
Sex (%)			
Female	173,748 (65.18)	28,958 (65.18)	144,790 (65.18)
Male	92,832 (34.82)	15,472 (34.82)	77,360 (34.82)
Educational levels (%)			
University	117,961 (44.25)	18,314 (41.22)	99,647 (44.86)
High school	31,330 (11.75)	5041 (11.35)	26,289 (11.83)
Middle school	74,168 (27.82)	12,761 (28.72)	61,407 (27.64)
Unknown	43,121 (16.18)	8314 (18.71)	34,807 (15.67)
Ethnicity (%)			
White	238,230 (89.37)	40,439 (91.02)	197,791 (89.03)
Others	26,880 (10.08)	3752 (8.44)	23,128 (10.41)
Unknown	1470 (0.55)	239 (0.54)	1231 (0.55)
BMI (%)			
< 18.5	1613 (0.61)	260 (0.59)	1353 (0.61)
18.5–25	94,173 (35.33)	13,906 (31.30)	80,267 (36.13)
25–30	107,227 (40.22)	17,825 (40.12)	89,402 (40.24)
> 30	62,008 (23.26)	12,174 (27.40)	49,834 (22.43)
Unknown	1559 (0.58)	265 (0.60)	1294 (0.58)
Smoking status (%)			
Never	151,147 (56.70)	22,962 (51.68)	128,185 (57.70)
Previous smokers	84,360 (31.65)	14,713 (33.12)	69,647 (31.35)
Current smokers	29,623 (11.11)	6499 (14.63)	23,124 (10.41)
Unknown	1450 (0.54)	256 (0.58)	1194 (0.54)
Townsend deprivation index (%)			
< − 3.6398	59,802 (22.43)	9967 (22.43)	49,835 (22.43)
− 3. 6398– − 2.1355	63,714 (23.90)	10,619 (23.90)	53,095 (23.90)
− 2.1355–0.5495	66,582 (24.98)	11,097 (24.98)	55,485 (24.98)
≥ 0. 5495	76,482 (28.69)	12,747 (28.69)	63,735 (28.69)
History of other psychiatric disorders (%)			
No	252,280 (94.64)	38,959 (87.69)	213,321 (96.03)
Yes	14,300 (5.36)	5471 (12.31)	8829 (3.97)
Family history of CVD (%)			
No	118,281 (44.37)	18,731 (42.16)	99,550 (44.81)
Yes	148,299 (55.63)	25,699 (57.84)	122,600 (55.19)
CVD polygenic risk score (%)			
Low	64,691 (24.27)	11,092 (24.97)	53,599 (24.13)
Intermediate	64,691 (24.27)	11,139 (25.07)	53,552 (24.11)
High	64,711 (24.27)	11,191 (25.19)	53,520 (24.09)
Unknown	72,487 (27.19)	11,008 (24.78)	61,479 (27.67)
CCI score (%)			
0	265,372 (99.55)	44,160 (99.39)	221,212 (99.58)
1	629 (0.24)	144 (0.33)	485 (0.22)
≥ 2	579 (0.22)	126 (0.28)	453 (0.20)
Any CVD (%)			
No	197,637 (74.14)	30,634 (68.95)	167,003 (75.18)
Yes	68,943 (25.86)	13,796 (31.05)	55,147 (24.82)

This table shows the basic characteristics of the patients with common psychiatric disorders and their matched unexposed individuals

*CCI* charlson comorbidity index; *SD*: standard deviation; *BMI* body mass index; *CVD* cardiovascular disease

^**1**^At most five participants who were alive, retained in the cohort and free of psychiatric disorders at the corresponding index date were individually matched to each individual with psychiatric disorders based on sex, and year of birth

During a mean follow-up time of 12.28 years, 13,796 (crude incidence rate: 25.34 per 1000 person-years) and 55,147 (20.20 per 1000 person-years) incident cases of CVD were identified among the exposed and matched unexposed individuals, respectively. The main attenuation of HRs was observed after the additional adjustment for BMI, smoking status, history of other psychiatric disorders, and CCI, from 1.29 [1.26–1.31] to 1.21 [1.18–1.22], while little attenuation occurred after additional adjustment for disease susceptibility to CVD (Table 2). The risk of CVD was higher for patients with common psychiatric disorders compared with matched unexposed individuals (HR [95% CIs]: 1.19 [1.17–1.22]), especially during the first six months of follow-up (1.72 [1.55–1.91], Table 2). All six major categories of CVD, as well as the defined acute cardiovascular events, showed similar risk patterns, with the strongest estimates noted for cerebrovascular disease (2.73 [1.81–4.14] for ≤ 6 months and 1.29 [1.21–1.38] for > 6 months, Table 2). Comparable results were found for anxiety, depression, and stress-related disorders, although estimates for some specific CVDs were marginally significant among stress-related disorders where a limited number of studied events were observed (Table S4). We obtained significantly higher HRs among individuals with an inpatient diagnosis of common psychiatric disorders than among those with only a self-reported/ primary care diagnosis (1.81 [1.65–1.98] vs 1.17 [1.15–1.20], *p for difference* < 0.0001, Table S5). Also, the magnitude of the studied associations was stronger among individuals with younger than among those with older diagnosis age of psychiatric disorders (Table S6, for the whole study period: HR = 1.25 [1.20–1.31] for < 47 years vs 1.18 [1.15–1.22] for > 55 years, *p for difference* = 0.0327, and for > 6 month follow-up: HR = 1.24 [1.19–1.30] vs 1.16 [1.13–1.20], *p for difference* = 0.0145).

**Table 2:** Hazard ratios (HRs) with 95% confidence intervals (CIs) of cardiovascular disease among patients with common psychiatric disorders compared to matched unexposed individuals

	The whole study period		≤ 6 months follow-up		> 6 months follow-up	
	No. of cases (incidence^1^) in patients/matched individuals	HR (95% CI)^2^	No. of cases (incidence^1^) in patients/matched individuals	HR (95% CI)^2^	No. of cases (incidence^1^) in patients/matched individuals	HR (95% CI)^2^
Model information						
Controlled for sex, year of birth, TDI, ethnicity and educational level	13,796 (25.34)/55,147 (20.20)	1.29 (1.26–1.31)	517 (23.75)/1411 (12.94)	1.81 (1.63–2.00)	13,279 (25.41)/52,919 (20.39)	1.27 (1.24–1.30)
As above + BMI + smoking status + history of other psychiatry disorders + CCI		1.21 (1.18–1.23)		1.73 (1.56–1.92)		1.19 (1.17–1.22)
As above + family history of CVDs		1.20 (1.18–1.23)		1.72 (1.55–1.91)		1.19 (1.16–1.21)
Subtypes of cardiovascular diseases^3^						
Hypertensive diseases	10,804 (19.09)/42,479 (15.12)	1.20 (1.17–1.22)	339 (15.54)/965 (8.84)	1.61 (1.42–1.84)	10,465 (19.24)/41,068 (15.31)	1.18 (1.16–1.21)
Ischemic heart disease	3,520 (5.74)/12,590 (4.23)	1.27 (1.22–1.32)	116 (5.30)/306 (2.80)	1.73 (1.38–2.18)	3404 (5.75)/12,206 (4.26)	1.26 (1.21–1.31)
Embolism and thrombosis	648 (1.02)/2512 (0.82)	1.14 (1.04–1.25)	16 (0.73)/38 (0.35)	2.14 (1.11–4.14)	632 (1.03)/2472 (0.84)	1.13 (1.03–1.23)
Arrhythmia/conduction disorder	2991 (4.80)/12,293 (4.10)	1.12 (1.07–1.17)	57 (2.60)/193 (1.77)	1.40 (1.03–1.90)	2934 (4.88)/12,081 (4.19)	1.11 (1.07–1.16)
Heart failure	846 (1.33)/3055 (1.00)	1.17 (1.08–1.27)	15 (0.69)/28 (0.26)	2.02 (0.92–4.41)	831 (1.36)/3025 (1.03)	1.17 (1.07–1.26)
Cerebrovascular disease	1324 (2.10)/4561 (1.50)	1.31 (1.23–1.40)	44 (2.01)/82 (0.75)	2.73 (1.81–4.14)	1280 (2.10)/4474 (1.53)	1.29 (1.21–1.38)
Acute cardiovascular events	1716 (2.73)/6594 (2.18)	1.15 (1.09–1.22)	56 (2.56)/146 (1.34)	1.77 (1.26–2.49)	1660 (2.74)/6433 (2.21)	1.14 (1.07–1.20)

^1^Per 1000 person-years

^2^Hazard ratios (with 95% confidence intervals) of cardiovascular disease in patients with common psychiatric disorders compared to matched unexposed individuals, derived from Cox regression models stratified by matching identifiers and adjusted for covariates listed in model information column

^3^Hazard ratios (with 95% confidence intervals) of subtypes of cardiovascular diseases in individual with common psychiatric disorders compared to sex, TDI, and year of birth matched unexposed individuals, derived from Cox regression models stratified by matching identifiers and adjusted for sex, year of birth, TDI, educational level, ethnicity, smoking status, BMI, history of other psychiatric disorders, family history of CVD, and CCI score. Definition of subtype's cardiovascular diseases by ICD-10 codes can be found in the Table S1 in the supplement

### The Role of Disease Susceptibility

We observed similar associations among individuals with and without a family history of CVD (1.73 [1.50–2.00] vs 1.88 [1.49–2.37], *p for difference* = 0.5506 within six months of follow-up, and 1.20 [1.16–1.23] vs 1.19 [1.14–1.24], *p for difference* = 0.7490 beyond six month follow-up, Table 3). Likewise, comparable risk elevations were observed among individuals with different levels of CVD PRS within six months of follow-up (e.g., for ischemic heart disease, 3.33 [1.13–9.79] and 1.73 [1.03–2.92] for low and high PRS, *p for difference* = 0.2843) and beyond (1.31 [1.14–1.51] and 1.21 [1.12–1.32] for low and high PRS, *p for difference* = 0.3391, Table 3).

**Table 3:** Hazard ratios (HRs) with 95% confidence intervals (CIs) of cardiovascular disease among patients with common psychiatric disorders compared to matched unexposed individuals, by different disease susceptibility

	≤ 6 month follow-up		> 6 month follow-up	
Disease susceptibility	No. of cases (incidence^1^) in patients/matched individuals	HR (95% CI)^2^	No. of cases (incidence^1^) in patients/matched individuals	HR (95% CI)^2^
Family history of cardiovascular disease				
No	171 (18.62)/213 (9.903)	1.88 (1.49–2.37)	4736 (21.01)/8134 (15.63)	1.19 (1.14–1.24)
Yes	346 (27.51)/548 (15.36)	1.73 (1.50–2.00)	8543 (28.74)/20,087 (23.94)	1.20 (1.16–1.23)
*p* for difference^3^		0.5506		0.7490
*p* for interaction^4^		0.7255		0.4865
Level of polygenic risk score (PRS) of the corresponding cardiovascular disease^5^				
Hypertensive diseases				
Low PRS (< the lower tertile)	67 (11.57)/45 (5.64)	1.72 (1.07–2.76)	2129 (14.28)/2297 (11.45)	1.18 (1.09–1.26)
Moderate PRS (lower–upper tertiles)	104 (16.88)/63 (7.53)	2.42 (1.62–3.64)	3011 (19.44)/3314 (16.1)	1.17 (1.10–1.24)
High PRS (> the upper tertile)	127 (20.26)/97 (11.52)	1.86 (1.37–2.54)	3912 (25.92)/4229 (20.89)	1.19 (1.13–1.26)
*p* for difference^3^		0.7864		0.8556
*p* for interaction^4^		0.9880		0.9606
Ischemic heart disease				
Low PRS (< the lower tertile)	16 (2.73)/7 (0.86)	3.33 (1.13–9.79)	577 (3.59)/553 (2.57)	1.31 (1.14–1.51)
Moderate PRS (lower–upper tertiles)	24 (3.89)/14 (1.68)	2.46 (1.11–5.46)	843 (4.98)/840 (3.82)	1.20 (1.07–1.35)
High PRS (> the upper tertile)	57 (9.14)/43 (5.17)	1.73 (1.03–2.92)	1544 (9.29)/1543 (7.12)	1.21 (1.12–1.32)
*p* for difference^3^		0.2843		0.3391
*p* for interaction^4^		0.9262		0.4360
Embolism and thrombosis				
Low PRS (< the lower tertile)	2 (0.33)/5 (0.6)	NA	134 (0.78)/125 (0.56)	1.26 (0.94–1.70)
Moderate PRS (lower–upper tertiles)	8 (1.3)/4 (0.48)	8.27 (1.48–46.18)	179 (1.04)/192 (0.86)	1.12 (0.87–1.44)
High PRS (> the upper tertile)	6 (0.99)/4 (0.49)	0.38 (0.03–4.92)	255 (1.5)/254 (1.15)	1.26 (1.03–1.53)
*p* for difference^3^		NA		1
*p* for interaction^4^		0.8070		0.8693
Arrhythmia/conduction disorder				
Low PRS (< the lower tertile)	6 (0.99)/11 (1.3)	0.65 (0.15–2.80)	554 (3.3)/637 (2.8)	1.13 (0.98–1.29)
Moderate PRS (lower–upper tertiles)	14 (2.3)/14 (1.7)	1.50 (0.55–4.09)	695 (4.2)/839 (3.8)	1.05 (0.93–1.18)
High PRS (> the upper tertile)	33 (5.3)/30 (3.6)	2.56 (1.30–5.05)	1389 (8.3)/1595 (7.2)	1.12 (1.03–1.22)
*p* for difference^3^		0.0959		0.9141
*p* for interaction^4^		0.1131		0.8893
Heart failure				
Low PRS (< the lower tertile)	1 (0.17)/1 (0.12)	NA	128 (0.76)/137 (0.62)	1.40 (1.04–1.90)
Moderate PRS (lower–upper tertiles)	1 (0.16)/0 (0)	NA	173 (1.01)/164 (0.74)	1.13 (0.86–1.47)
High PRS (> the upper tertile)	11 (1.76)/6 (0.72)	3.51 (1.03–11.99)	434 (2.49)/437 (1.93)	1.13 (0.95–1.33)
*p* for difference^3^		NA		0.2237
*p* for interaction^4^		NA		0.4423
Cerebrovascular disease				
Low PRS (< the lower tertile)	5 (0.83)/6 (0.73)	1.10 (0.12–10.33)	279 (1.66)/272 (1.23)	1.28 (1.05–1.56)
Moderate PRS (lower–upper tertiles)	11 (1.81)/3 (0.36)	NA	346 (2.03)/289 (1.3)	1.53 (1.27–1.85)
High PRS (> the upper tertile)	19 (3.07)/12 (1.43)	2.30 (0.97–5.46)	523 (3.04)/562 (2.5)	1.13 (0.98–1.31)
*p* for difference^3^		0.5451		0.3196
*p* for interaction^4^		0.0789		0.1086

^1^Per 1000 person-years

^2^Cox regression models were stratified by matching identifiers and adjusted for sex, TDI, year of birth, educational level, ethnicity, smoking status, BMI, history of other psychiatry disorders, and CCI score

^3^The differences in hazard ratios for family history of CVD were assessed by Wald test. The differences in hazard ratios for CVD PRS were assessed between low and high subgroups by Wald test

^4^The *p* for interaction for family history of CVD was assessed by introducing an interaction term to the Cox models. The *p* for interaction for CVD PRS was assessed by introducing an interaction term of low and high subgroups to the Cox models

^5^Polygenic risk scores of the corresponding cardiovascular diseases were calculated based on published GWAS summary statistics data

### Disease Trajectory Analysis

**Fig. 2:**
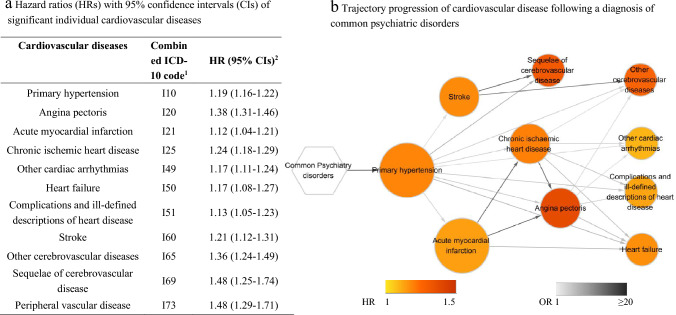
Significant individual cardiovascular diseases and its trajectory progression subsequent to a diagnosis of common psychiatric disease **a** Hazard ratios (HRs) with 95% confidence intervals (CIs) of significant individual cardiovascular diseases among patients with common psychiatric disorders compared to matched unexposed individuals. ^1^Combined ICD-10 code; mapping between the original ICD-10 code and the combined ICD-10 code can be found in Table S2; ^2^Hazard ratios (with 95% confidence intervals) of different cardiovascular diseases more in patients with common psychiatric disorders compared to matched individuals without psychiatric disorders, derived from Cox regression models stratifying by matching identifiers and adjusted for age, year of birth, TDI, educational level, ethnicity, smoking status, BMI, history of other psychiatric disorders, family history of CVD, and CCI score. **b** Trajectory progression of cardiovascular disease following a diagnosis of common psychiatric disorders. This figure illustrates following trajectory progression of cardiovascular disease identified in our analysis. The combined cardiovascular diseases are shown within the circle. The color of the nodes represents the hazard ratios of this cardiovascular disease when comparing patients with common psychiatric disorders to matched unexposed individuals. The color of the arrows indicates the odds ratio of the sequential association between the two cardiovascular disease events among patients with common psychiatric disorders

Among 26 specific CVDs, 19 CVDs with more than 200 cases among patients with common psychiatric disorders were involved in the association analysis (Fig. S3). Among these, 11 CVDs showed significant associations with a prior diagnosis of common psychiatric disorders after multiple testing (Fig. 2a), forming 110 CVD pairs and 43 of them passed the threshold of prevalence (i.e., co-occurred among at least 100 individuals). Finally, the nested case–control analyses confirmed the associations of 23 CVD pairs, consisting of 10 CVDs, which then contributed to the visualized trajectory networks (Fig. 2a, Fig. S3 and Table S7). Figure 2b shows the CVD trajectory network subsequent to the diagnosis of common psychiatric diseases, which originate from primary hypertension, acute myocardial infarction, and stroke located at the first and second layers, which are further connected to a wide range of other CVDs.

In the sensitivity analysis, defining the CVD cases solely by primary diagnoses, instead of all diagnoses, in UK Biobank inpatient data led to largely similar estimates (Tables S8-S10). However, the number of CVDs involved in the trajectory network was substantially reduced due to insufficient data power, where we had only three CVD pairs in the disease tree with an initial node of acute myocardial infarction (Fig. S4 and S5).

## Discussion

In this matched cohort study of the UK Biobank, we found that individuals with common psychiatric disorders (i.e., anxiety, depression, and stress-related disorders) were at elevated risk of multiple CVDs, especially within the first six months of follow-up. With mutually verified results obtained from stratified analyses by family history of CVD and by CVD PRS, the observed associations seemed to be constant across individuals with different predispositions to CVD. Furthermore, disease trajectory analysis indicated that primary hypertension, acute myocardial infarction, and stroke were the CVD types first affected by a diagnosis of common psychiatric disorders, underscoring their key roles in the progression path from prior psychiatric disorders to downstream CVD sequelae. Therefore, timely interventions that target these specific CVDs might effectively prevent or interrupt the overall risk of CVD among individuals with common psychiatric disorders.

In line with our results, multiple large-scale cohort studies have reported an association between a diagnosis of psychiatric disorders and a subsequently elevated risk of CVD, despite often insufficient control of important confounders, such as lifestyle (Stein et al. 2014; Song et al. 2019; Wium-Andersen et al. 2019; Han et al. 2021) and familial factors (Yu et al. 2010; Stein et al. 2014; Swain et al. 2015; Kollia et al. 2017; Momen et al. 2020; Han et al. 2021). Our finding that the association was not modified by family history of CVD gains support from two recent Danish twin studies (Wium-Andersen et al. 2019, 2020), both of which concluded that genetic factors and early-life environment did not explain the association of depression with ischemic heart disease and stroke using a study sample of 6714 twins (Wium-Andersen et al. 2019) and 63,038 twins (Wium-Andersen et al. 2020), respectively. The Nurses' Health Study II and the Normative Aging Study also revealed no attenuation in the magnitude of association between psychiatric disorders and CVD after additional adjustment for family history of CVD (Kubzansky et al. 2007; Sumner et al. 2015). Our analysis on CVD PRS expanded this knowledgebase further and, for the first time, showed similar associations across groups with different levels of disease susceptibility to CVD. Taken together, these results underscore the importance of surveillance for CVD events among individuals with common psychiatric disorders, regardless of disease liability.

Based on the disease trajectory network, we identified three specific CVDs (i.e., primary hypertension, followed by acute myocardial infarction, and stroke) placed at the first two layers, indicating that they were earliest affected by a prior diagnosis of common psychiatric disorders. Although there are no existing studies on CVD trajectory networks after common psychiatric disorders, the direct effect of psychiatric disorders on these specifically CVDs has been implied in the previous studies. Mendelian randomization analyses showed associations of genetic predisposition to depression with ischemic stroke (Cai et al. 2019) and myocardial infarction (Lu et al. 2021), suggesting a causal link between depression and these CVDs. Also, the findings of experimental studies indicated direct physiological responses to mental stress, including endothelial dysfunction (monkeys (Strawn et al. 1991) and human (Ghiadoni et al. 2000)), dysregulation of blood pressure (Nas et al. 2020), and activation of the HPA axis (Jokinen and Nordstrom 2009). Moreover, similar downstream CVDs (e.g., chronic ischemic heart diseases, other cerebrovascular diseases, and other cardiac arrhythmias) after primary hypertension, and acute myocardial infarction can be identified using a disease trajectory browser that was developed based on national data from inpatient wards, outpatient clinics, and emergency room visits of Denmark (Siggaard et al. 2020). Collectively, these data suggest that these key CVDs (i.e., primary hypertension, acute myocardial infarction, and stroke) may be potential targets for preventing overall CVD risk or interrupting the development of further CVDs (i.e., diagnoses placed at later layers of the trajectory network) among individuals with psychiatric disorders.

The major strength of our study includes the multidimensional data from the UK Biobank, i.e., rich phenotypic information on family history of CVD, health-related outcomes, and individual-level genotype information, which enabled a comprehensive assessment of the modification role of CVD susceptibility on the studied associations and the temporal patterns of CVD development following a diagnosis of common psychiatric disorders. These rich data sources also enabled vigorous control of a wide range of important confounders, including sociodemographic and behavioral factors, as well as baseline health status. Other strengths include the long and almost complete follow-up (mean follow-up time of 12.28 years), providing sufficient surveillance period for CVD outcomes, and the independent collection of diagnoses for psychiatric disorders and CVD, which minimizes the risk of information bias.

Our study has several limitations. First, the findings in our study should be validated in an independent cohort in future studies. Second, as both primary and secondary diagnoses from UK Biobank inpatient hospital data were used for CVD identification, and a proportion of psychiatric disorders were merely ascertained by diagnoses in self-reported and primary care data, the incident date for some mild forms of cases (both psychiatric disorders and CVDs) may not be accurate, leading to a risk of reverse causality. Consequently, the appearance of primary hypertension as the initial node in the CVD trajectory tree of the main analysis may imply the importance of primary hypertension as a comorbidity of psychiatric disorders on other subsequent CVDs, instead of a key progression pathway. Nevertheless, our analyses on more severe psychiatric disorders (i.e., inpatient diagnosis) and acute cardiovascular events should be less affected by such concern. Third, despite our efforts to detect the role of disease severity on the studied associations by subgrouping exposed patients according to the source of their psychiatric disorder diagnosis (i.e., inpatient vs only self-reported/primary care diagnosis), the lack of detailed data on onset symptoms, progress, and treatment of these common psychiatric disorders precluded further discussions on many relevant questions, such as to what extent the delays in diagnosis of psychiatric disorders (i.e., a long time interval between disease onset and receiving diagnosis) have biased our estimates, and if the use of psychotropic medication could affect the observed associations. Fourth, the SNPs used in the calculation for CVD PRS were obtained from GWAS studies which mainly focused on coronary arterial diseases, not all CVDs. However, a common genetic basis has been demonstrated between different CVDs (Dichgans et al. 2014; Jafaripour et al. 2019), and the generated PRS has been confirmed to be associated with the CVD phenotype of our dataset in our validation step. Furthermore, we obtained largely similar result patterns in the analyses of six major categories of CVDs. Fifth, as the disease trajectory analysis is a data-driven approach which requires a large sample size, our analysis might have missed some rare CVDs in the trajectory networks after psychiatric disorders. Further, because some important covariates, such as BMI and smoking status, were measured at baseline, they might not accurately reflect the real status of these factors at the time of psychiatric disorder diagnosis, resulting in a possibility of adjusting for mediators (i.e., occurred between the studied exposure and outcome), not confounders, and thereby underestimated association. Finally, generalization of our findings to the entire UK population, or other populations, should be done with caution because the UK Biobank population is older (only 40- to 69-year-old residents were invited to join) and generally healthier than the general UK population (Jones 2013; Sudlow et al. 2015).

## Conclusion

In conclusion, individuals with common psychiatric disorders have an elevated risk of CVD hospitalization regardless of their predisposition to CVD. Hypertension, acute myocardial infarction, and stroke represent the three initial CVDs that link psychiatric disorders to further CVDs, highlighting a need of timely interventions that target those specific CVDs to prevent overall CVD risk among individuals with common psychiatric disorders.

## Supplementary Information

Below is the link to the electronic supplementary material.

Supplementary file1 (DOCX 16 KB)

Supplementary file2 (DOCX 424 KB)

## Data Availability

Source data for Fig. 2b have been provided in the supplementary material (Table S7). Original Data are held by UK Biobank Limited. Due to the ethical approval of the UK Biobank from the NHS National Research Ethics Service and the ethical approval of the current study, we cannot make the data publicly available. However, all researchers can access the data upon making an application to the UK Biobank. Detailed information on data application can be found at http://www.ukbiobank.ac.uk/.

## Abbreviations

CVD: Cardiovascular disease
WHO: World Health Organization
HPA: Hypothalamic–pituitary–adrenal
GWAS: Genome-wide association studies
SNP: Single-nucleotide polymorphism
ICD: International Classification of Diseases
PPV: Positive predictive value
PRS: Polygenic risk score
TDI: Townsend deprivation index
BMI: Body mass index
CCI: Charlson Comorbidity Index
HR: Hazard ratios
CI: Confidence interval
OR: Odds ratio

## References

[CR1] Cai H, Cai B, Zhang H et al (2019) Major depression and small vessel stroke: a Mendelian randomization analysis. J Neurol 266(11):2859–2866. 10.1007/s00415-019-09511-w 31435769 10.1007/s00415-019-09511-w

[CR2] The Emerging Risk Factors Collaboration (2015) Association of cardiometabolic multimorbidity with mortality. JAMA 314(1):52–60. 10.1001/jama.2015.7008 26151266 10.1001/jama.2015.7008 PMC4664176

[CR3] Consortium CAD, Deloukas P, Kanoni S et al (2013) Large-scale association analysis identifies new risk loci for coronary artery disease. Nat Genet 45(1):25–33. 10.1038/ng.2480 23202125 10.1038/ng.2480 PMC3679547

[CR4] DALYs GBD, Collaborators H, Murray CJ et al (2015) Global, regional, and national disability-adjusted life years (DALYs) for 306 diseases and injuries and healthy life expectancy (HALE) for 188 countries, 1990–2013: quantifying the epidemiological transition. Lancet 386(10009):2145–2191. 10.1016/S0140-6736(15)61340-X 26321261 10.1016/S0140-6736(15)61340-X PMC4673910

[CR5] Davis KAS, Bashford O, Jewell A et al (2018) Using data linkage to electronic patient records to assess the validity of selected mental health diagnoses in English Hospital Episode Statistics (HES). PLoS ONE 13(3):e0195002. 10.1371/journal.pone.0195002 29579109 10.1371/journal.pone.0195002 PMC5868851

[CR6] Dichgans M, Malik R, Konig IR et al (2014) Shared genetic susceptibility to ischemic stroke and coronary artery disease: a genome-wide analysis of common variants. Stroke 45(1):24–36. 10.1161/STROKEAHA.113.002707 24262325 10.1161/STROKEAHA.113.002707 PMC4112102

[CR7] Forsdahl A (1979) Are poor living conditions in childhood and adolescence and important risk factor for arteriosclerotic heart disease? Int J Rehabil Res 2(2):238–239. 10.1097/00004356-197905000-00008 478740 10.1097/00004356-197905000-00008

[CR8] Ghiadoni L, Donald AE, Cropley M et al (2000) Mental stress induces transient endothelial dysfunction in humans. Circulation 102(20):2473–2478. 10.1161/01.cir.102.20.2473 11076819 10.1161/01.cir.102.20.2473

[CR9] Han X, Hou C, Yang H et al (2021) Disease trajectories and mortality among individuals diagnosed with depression: a community-based cohort study in UK Biobank. Mol Psychiatry. 10.1038/s41380-021-01170-6 34035478 10.1038/s41380-021-01170-6 PMC8145187

[CR10] Holt JD, Prentice RL (1974) Survival analyses in twin studies and matched pair experiments. Biometrika 61:17–30. 10.1093/biomet/61.1.17

[CR11] Iannotti RJ, Zuckerman AE, Rifai N (2000) Correlations of cardiovascular disease risk factors between African American siblings. J Pediatr 136(4):511–519. 10.1016/s0022-3476(00)90015-5 10753250 10.1016/s0022-3476(00)90015-5

[CR12] Jafaripour S, Sasanejad P, Dadgarmoghaddam M et al (2019) ADAMTS7 and ZC3HC1 share genetic predisposition to coronary artery disease and large artery ischemic stroke. Crit Rev Eukaryot Gene Expr 29(4):351–361. 10.1615/CritRevEukaryotGeneExpr.2019028209 31679296 10.1615/CritRevEukaryotGeneExpr.2019028209

[CR13] Janszky I, Ahnve S, Lundberg I et al (2010) Early-onset depression, anxiety, and risk of subsequent coronary heart disease: 37-year follow-up of 49,321 young Swedish men. J Am Coll Cardiol 56(1):31–37. 10.1016/j.jacc.2010.03.033 20620714 10.1016/j.jacc.2010.03.033

[CR14] John A, McGregor J, Fone D et al (2016) Case-finding for common mental disorders of anxiety and depression in primary care: an external validation of routinely collected data. BMC Med Inform Decis Mak 16:35. 10.1186/s12911-016-0274-7 26979325 10.1186/s12911-016-0274-7 PMC4791907

[CR15] Jokinen J, Nordstrom P (2009) HPA axis hyperactivity and cardiovascular mortality in mood disorder inpatients. J Affect Disord 116(1–2):88–92. 10.1016/j.jad.2008.10.025 19054568 10.1016/j.jad.2008.10.025

[CR16] Jones PB (2013) Adult mental health disorders and their age at onset. Br J Psychiatry Suppl 54:s5-10. 10.1192/bjp.bp.112.119164 23288502 10.1192/bjp.bp.112.119164

[CR17] Khandaker GM, Zuber V, Rees JMB et al (2020) Shared mechanisms between coronary heart disease and depression: findings from a large UK general population-based cohort. Mol Psychiatry 25(7):1477–1486. 10.1038/s41380-019-0395-3 30886334 10.1038/s41380-019-0395-3 PMC7303009

[CR18] Kivimaki M, Batty GD, Singh-Manoux A et al (2017) Validity of cardiovascular disease event ascertainment using linkage to UK hospital records. Epidemiology 28(5):735–739. 10.1097/EDE.0000000000000688 28570383 10.1097/EDE.0000000000000688 PMC5540351

[CR19] Kollia N, Panagiotakos D, Georgousopoulou E et al (2017) Exploring the path between depression, anxiety and 10-year cardiovascular disease incidence, among apparently healthy Greek middle-aged adults: The ATTICA study. Maturitas 106:73–79. 10.1016/j.maturitas.2017.09.005 29150168 10.1016/j.maturitas.2017.09.005

[CR20] Kubzansky LD, Koenen KC, Spiro A 3rd et al (2007) Prospective study of posttraumatic stress disorder symptoms and coronary heart disease in the Normative Aging Study. Arch Gen Psychiatry 64(1):109–116. 10.1001/archpsyc.64.1.109 17199060 10.1001/archpsyc.64.1.109

[CR21] Laurent S, Boutouyrie P (2015) The structural factor of hypertension: large and small artery alterations. Circ Res 116(6):1007–1021. 10.1161/CIRCRESAHA.116.303596 25767286 10.1161/CIRCRESAHA.116.303596

[CR22] Lee J, Joo EJ, Lim HJ et al (2015) Proteomic analysis of serum from patients with major depressive disorder to compare their depressive and remission statuses. Psychiatry Investig 12(2):249–259. 10.4306/pi.2015.12.2.249 25866527 10.4306/pi.2015.12.2.249 PMC4390597

[CR23] Lu Y, Wang Z, Georgakis MK et al (2021) Genetic liability to depression and risk of coronary artery disease, myocardial infarction, and other cardiovascular outcomes. J Am Heart Assoc 10(1):e017986. 10.1161/JAHA.120.017986 33372528 10.1161/JAHA.120.017986 PMC7955472

[CR24] Marees AT, de Kluiver H, Stringer S et al (2018) A tutorial on conducting genome-wide association studies: Quality control and statistical analysis. Int J Methods Psychiatr Res 27(2):e1608. 10.1002/mpr.1608 29484742 10.1002/mpr.1608 PMC6001694

[CR25] Momen NC, Plana-Ripoll O, Agerbo E et al (2020) Association between mental disorders and subsequent medical conditions. N Engl J Med 382(18):1721–1731. 10.1056/NEJMoa1915784 32348643 10.1056/NEJMoa1915784 PMC7261506

[CR26] Nas Z, Riese H, van Roon AM et al (2020) Higher anxiety is associated with lower cardiovascular autonomic function in female twins. Twin Res Hum Genet 23(3):156–164. 10.1017/thg.2020.47 32539904 10.1017/thg.2020.47

[CR27] NINDS Stroke Genetics Network (SiGN), International Stroke Genetics Consortium (ISGC) (2016) Loci associated with ischaemic stroke and its subtypes (SiGN): a genome-wide association study. Lancet Neurol 15(2):174–184. 10.1016/S1474-4422(15)00338-5 26708676 10.1016/S1474-4422(15)00338-5 PMC4912948

[CR28] Nikpay M, Goel A, Won HH et al (2015) A comprehensive 1,000 Genomes-based genome-wide association meta-analysis of coronary artery disease. Nat Genet 47(10):1121–1130. 10.1038/ng.3396 26343387 10.1038/ng.3396 PMC4589895

[CR29] Prive F, Arbel J, Vilhjalmsson BJ (2020) LDpred2: better, faster, stronger. Bioinformatics. 10.1093/bioinformatics/btaa1029 33326037 10.1093/bioinformatics/btaa1029 PMC8016455

[CR30] Quan H, Sundararajan V, Halfon P et al (2005) Coding algorithms for defining comorbidities in ICD-9-CM and ICD-10 administrative data. Med Care 43(11):1130–1139. 10.1097/01.mlr.0000182534.19832.83 16224307 10.1097/01.mlr.0000182534.19832.83

[CR31] Siggaard T, Reguant R, Jorgensen IF et al (2020) Disease trajectory browser for exploring temporal, population-wide disease progression patterns in 7.2 million Danish patients. Nat Commun 11(1):4952. 10.1038/s41467-020-18682-4 33009368 10.1038/s41467-020-18682-4 PMC7532164

[CR32] Song H, Fang F, Arnberg FK et al (2019) Stress related disorders and risk of cardiovascular disease: population based, sibling controlled cohort study. BMJ 365:l1255. 10.1136/bmj.l1255 30971390 10.1136/bmj.l1255 PMC6457109

[CR33] Stein DJ, Aguilar-Gaxiola S, Alonso J et al (2014) Associations between mental disorders and subsequent onset of hypertension. Gen Hosp Psychiatry 36(2):142–149. 10.1016/j.genhosppsych.2013.11.002 24342112 10.1016/j.genhosppsych.2013.11.002 PMC3996437

[CR34] Strawn WB, Bondjers G, Kaplan JR et al (1991) Endothelial dysfunction in response to psychosocial stress in monkeys. Circ Res 68(5):1270–1279. 10.1161/01.res.68.5.1270 2018991 10.1161/01.res.68.5.1270

[CR35] Sudlow C, Gallacher J, Allen N et al (2015) UK biobank: an open access resource for identifying the causes of a wide range of complex diseases of middle and old age. PLoS Med 12(3):e1001779. 10.1371/journal.pmed.1001779 25826379 10.1371/journal.pmed.1001779 PMC4380465

[CR36] Sumner JA, Kubzansky LD, Elkind MS et al (2015) Trauma exposure and posttraumatic stress disorder symptoms predict onset of cardiovascular events in women. Circulation 132(4):251–259. 10.1161/CIRCULATIONAHA.114.014492 26124186 10.1161/CIRCULATIONAHA.114.014492 PMC4519406

[CR37] Swain NR, Lim CC, Levinson D et al (2015) Associations between DSM-IV mental disorders and subsequent non-fatal, self-reported stroke. J Psychosom Res 79(2):130–136. 10.1016/j.jpsychores.2015.05.008 26094010 10.1016/j.jpsychores.2015.05.008 PMC4621960

[CR38] Tao H, Chen X, Zhou H et al (2020) Changes of serum melatonin, interleukin-6, homocysteine, and complement C3 and C4 levels in patients with depression. Front Psychol 11:1271. 10.3389/fpsyg.2020.01271 32655450 10.3389/fpsyg.2020.01271 PMC7324806

[CR39] Townsend P, Phillimore P, Beattie A (1988) Health and deprivation: inequality and the North. Routledge. 10.4324/9781003368885

[CR40] Weinberger AH, Kashan RS, Shpigel DM et al (2017) Depression and cigarette smoking behavior: a critical review of population-based studies. Am J Drug Alcohol Abuse 43(4):416–431. 10.3109/00952990.2016.1171327 27286288 10.3109/00952990.2016.1171327

[CR41] Whiteford HA, Degenhardt L, Rehm J et al (2013) Global burden of disease attributable to mental and substance use disorders: findings from the Global Burden of Disease Study 2010. Lancet 382(9904):1575–1586. 10.1016/S0140-6736(13)61611-6 23993280 10.1016/S0140-6736(13)61611-6

[CR42] Wium-Andersen MK, Wium-Andersen IK, Jorgensen MB et al (2019) The association between depressive mood and ischemic heart disease: a twin study. Acta Psychiatr Scand 140(3):265–274. 10.1111/acps.13072 31306494 10.1111/acps.13072 PMC8039049

[CR43] Wium-Andersen MK, Villumsen MD, Wium-Andersen IK et al (2020) The familial and genetic contribution to the association between depression and cardiovascular disease: a twin cohort study. Mol Psychiatry. 10.1038/s41380-020-00954-6 33219357 10.1038/s41380-020-00954-6

[CR44] Woodfield R, Grant I, Group UKBSO et al (2015) Accuracy of Electronic Health Record Data for Identifying Stroke Cases in Large-Scale Epidemiological Studies: A Systematic Review from the UK Biobank Stroke Outcomes Group. PLoS ONE 10(10):e0140533. 10.1371/journal.pone.0140533 26496350 10.1371/journal.pone.0140533 PMC4619732

[CR45] Yang H, Pawitan Y, He W et al (2019) Disease trajectories and mortality among women diagnosed with breast cancer. Breast Cancer Res 21(1):95. 10.1186/s13058-019-1181-5 31420051 10.1186/s13058-019-1181-5 PMC6698019

[CR46] Yu RH, Ho SC, Lam CW et al (2010) Psychological factors and subclinical atherosclerosis in postmenopausal Chinese women in Hong Kong. Maturitas 67(2):186–191. 10.1016/j.maturitas.2010.06.014 20638205 10.1016/j.maturitas.2010.06.014

[CR47] Zorn JV, Schur RR, Boks MP et al (2017) Cortisol stress reactivity across psychiatric disorders: a systematic review and meta-analysis. Psychoneuroendocrinology 77:25–36. 10.1016/j.psyneuen.2016.11.036 28012291 10.1016/j.psyneuen.2016.11.036

