# Supplementary material for: Risk of Cardiovascular Disease Hospitalization After Common Psychiatric Disorders: Analyses of Disease Susceptibility and Progression Trajectory in the UK Biobank
Source: Phenomics. 2024 Jul 8;4(4):327–38. doi: 10.1007/s43657-023-00134-w (PMC11584824; doi:10.1007/s43657-023-00134-w)
Supplement: Supplementary file 1 — Supplementary file1 (DOCX 16 KB) [file 43657_2023_134_MOESM1_ESM.docx]

**Disease trajectory analyses**

**Step 1**: The first step is to identify the risks of all 26 specific cardiovascular diseases (CVDs) among individuals with common psychiatric diseases compared to matched unexposed individuals. Cox regression models were conducted, stratified by matched identifiers (unique ID for each exposed patient and their individually matched unexposed individuals), and adjusted for sex, birth year, Townsend deprivation index (TDI), educational levels, ethnicity, smoking status, BMI, history of other psychiatric disorders, family history of CVD, and CCI. To ensure statistical power, we limited the analyses to CVDs that occurred in at least 200 individuals with common psychiatric diseases. In each Cox analysis investigating the outcome CVD, a sub-cohort was formed by removing individuals with a previous history of the outcome CVD before follow-up started (i.e., diagnosis date of common psychiatric disorders), and follow-up of the individuals in the sub-cohort ended on date of the outcome CVD diagnosis, date of death, date of loss to follow-up or the end date of the study, whichever came first. To account for multiple testing, the p-value for statistical significance was set to 0.05/number of analyses performed (Bonferroni corrections). Only CVDs with hazard ratio (HR) > 1 and p < Bonferroni corrected threshold were considered in the second step.

**Step 2**: The second step is to identify the temporal order of CVD 1 and CVD 2 pairs (i.e., CVD 2 occurred after CVD 1) among individuals with common psychiatric diseases. Among these significant CVDs from the last step, we constructed all possible CVD 1 and CVD2 pairs. For each CVD1→CVD2 pair that was experienced by at least 100 individuals with common psychiatric diseases (200/2), binomial test was conducted to investigate whether significantly more individuals with common psychiatric diseases (>50%) had CVD2 diagnosed after CVD1 among those with both CVD1 and CVD2 diagnoses. Only CVD1→CVD2 pairs with binomial test p-value<Bonferroni corrected threshold were forwarded to the third step.

**Step 3**: The third step is to confirm the associations of CVD1→CVD2 pairs. Nested case-control study design and conditional logistic regression analyses were used to assess the magnitude of the associations. For each CVD1→CVD2 pair, we identified CVD2 as outcome and CVD1 as exposure. In each case-control dataset, at most 2 CVD2-free individuals at the same time were matched to each CVD2 case by sex, TDI, year of birth, and year of diagnosis of common psychiatric diseases. Conditional logistic regression was used to contrast the CVD2 odds of having a prior D1 diagnosis. Only CVD pairs with confirmed associations (i.e., Odds ratio [OR] > 1 and p < Bonferroni corrected threshold in the nested case-control analyses) were included to the construction of trajectory network.
