# Supplementary material for: Risk of Cardiovascular Disease Hospitalization After Common Psychiatric Disorders: Analyses of Disease Susceptibility and Progression Trajectory in the UK Biobank
Source: Phenomics. 2024 Jul 8;4(4):327–38. doi: 10.1007/s43657-023-00134-w (PMC11584824; doi:10.1007/s43657-023-00134-w)
Supplement: Supplementary file 2 — Supplementary file2 (DOCX 424 KB) [file 43657_2023_134_MOESM2_ESM.docx]

**Supplementary tables and figures**

**Table S1** International Classification of Diseases (ICD) codes and primary care codes for diseases identifications

**Table S2** Mapping between ICD-10 codes and Combined ICD codes for PheWAS analysis

**Table S3** Information of GWAS summary statistics for six major subtypes of cardiovascular diseases

**Table S4** Hazard ratios (HRs) with 95% confidence intervals (CIs) of cardiovascular disease among patients with common psychiatric disorders compared to matched unexposed individuals, by different psychiatric disorders

**Table S5** Hazard ratios (HRs) with 95% confidence intervals (CIs) of cardiovascular disease among patients with common psychiatric disorders compared to matched unexposed individuals, by different diagnosis source of common psychiatric disorders

**Table S6** Hazard ratios (HRs) with 95% confidence intervals (CIs) of cardiovascular disease among patients with common psychiatric disorders compared to matched unexposed individuals, by different age of psychiatric disorders diagnosis

**Table S7** Odds ratios (ORs) with 95% confidence intervals (CIs) for the significant pairs of cardiovascular diseases following a diagnosis of common psychiatric disorders

**Table S8** Hazard ratios (HRs) with 95% confidence intervals (CIs) of primary diagnosis of cardiovascular disease among patients with common psychiatric disorders compared to matched unexposed individuals

**Table S9** Hazard ratios (HRs) with 95% confidence intervals (CIs) of primary diagnosis of cardiovascular disease among patients with common psychiatric disorders compared to matched unexposed individuals, by different disease susceptibility

**Table S10** Odds ratios (ORs) with 95% confidence intervals (CIs) for the significant pairs of primary diagnosis of cardiovascular diseases following a diagnosis of common psychiatric disorders

**Fig. S1** Flowchart of selection on participants and SNPs for PRS calculation

**Fig. S2** Hazard ratios (95% CIs) of cardiovascular disease among patients with common psychiatric disorders compared with their matched unexposed individuals, stratified by time of follow-up

**Fig. S3** Flow chart of identifying trajectory progression of cardiovascular disease following a diagnosis of common psychiatric disorders

**Fig. S4** Flow chart of identifying trajectory progression of primary diagnosis of cardiovascular disease following a diagnosis of common psychiatric disorders

**Fig. S5** Trajectory progression of primary diagnosis of cardiovascular disease following a diagnosis of common psychiatric disorders

## **Table S1** International Classification of Diseases (ICD) codes and primary care codes for diseases identifications

|  | **ICD-9** | **ICD-10** | **Primary care READ V2 code** | **Primary care READ V3 code** |
| --- | --- | --- | --- | --- |
| **Anxiety** | 3000, 3002 | F40, F41 | E2000, E2001, E2002, E2004, E2005, E200z, E202., E2020, E2021, E2022, E2023, E2024, E2025, E2026, E2027, E2028, E2029, E202A,E202B, E202C, E202D, E202E, E202z, Eu40., Eu400, Eu401, Eu402, Eu403, Eu40y, Eu40z, Eu41., Eu410, Eu411, Eu412, Eu413, Eu41y, Eu41z | E200., E2000, E2002, E2004, E200z, E2020, E2021, E2022, E2023, E2024, E2025, E2026, E2027, E2028, E2029, E202E, Eu40., Eu400, Eu402, Eu40y, Eu40z, Eu41., Eu410, Eu413, Eu41y, Eu41z, Eu931, Ua1qa, Ua1qc, Ua1qd, Ua1qe, Ua1qf, Ua1qg, Ua1qh, Ua1qi, Ua1qj, Ua1qk, Ua1ql, , Ua1qm, Ua1qn, Ua1qo, Ua1qp, Ua1qs, Ua1qt, Ua1qU, Ua1qV, Ua1qW, Ua1qX, Ua1qY, X00RP, X00Sa, X00Sb, X00Sc, X00Sd, X00Se, X00Sr, X00SV, X00SW, X00SX, X00SY, X00SZ, X50G2, X50G3, X50G5, X50G6, X50GI, X75YV, X761d, X761n, X761q, X761t, X761u, X761y, X7627, X7628, X7629, X762a, X762C, X762E, X762F, X762G, X762H, X762T, X762Z, X78wp, Xa00r, Xa00s, Xa1a8, Xa1Ev, Xa3Vj, Xa3Vk, Xa3Vl, Xa3WH, Xa3WI, Xa3WJ, Xa7k9, Xa7kB, XaIo7, XaKVA, XE0rb, XE1Y7, XE1YA, XE1YB, XE1Zj, XM0Ak |
| **Depression** | 2961 | F32, F33 | E112., E1120, E1121, E1122, E1123, E1124, E1125, E1126, E112z, E113., E1130, E1131, E1132, E1133, E1134, E1135, E1136, E1137, E113z, E135., Eu32., Eu320, Eu321, Eu322, Eu323, Eu324, Eu325, Eu326, Eu327, Eu328, Eu329, Eu32A, Eu32B, Eu32y, Eu32z, Eu33., Eu330, Eu331, Eu332, Eu333, Eu334, Eu33y, Eu33z | 2257, E0043, E1120, E1121, E1122, E1123, E1124, E1125, E1126, E112z, E1130, E1131, E1132, E1133, E1134, E1135, E1136, E1137, E113z, E11y2, E130, E2B0., E2B1., Eu320, Eu321, Eu322, Eu323, Eu32y, Eu32z, Eu330, Eu331, Eu332, Eu333, Eu334, Eu33y, Eu33z, X00SO, X00SQ, X00SS, X00SU, Xa0wV, XaB9J, XaCHo, XaCHr, XaCHs, XaCIs, XaCIt, XaCIu, XaX53, XaX54, XaY2C, XE1Y0, XE1Y1, XE1YC, XE1Za, XE1Zb, XE1Zc, XE1Zd, XE1Ze, XE1Zf, XE1ZY, XE1ZZ, XM1GC, XSEGJ, XSGok, XSGol, XSGom, XSGon |
| **Stress-related disorders** | 308, 309 | F43 | E280., E281., E282., E283., E2830, E2831, E283z, E284., E28z., E2900, E2925, E292y, E292z, E293., E2930, E2931, E2932, E293z, E294., E29y., E29y1, E29y2, E29y4, E29yz, E29z., Eu43., Eu430, Eu431, Eu432, Eu433, Eu434, Eu435, Eu43y, Eu43z | 1B1L., 1BE.., E280., E281., E282., E283., E2830, E2831, E283z, E284., E29.., E290., E290z, E291., E292., E2920, E2921, E2922, E2924, E2925, E292y, E292z, E293., E2930, E2931, E2932, E293z, E294., E29y., E29y0, E29y1, E29y2, E29y3, E29y4, E29y5, E29yz, E29z., Eu430, Eu432, Eu43y, Eu43z, Eu930, Ry15., Ua18k, Ua18L, Ub1T9, X00Sf, X00TT, X40Js, Xa028, Xa18j, Xa18v, XaC2u, XaX55, XaX56, XaX58, XE1Ym, XE1Yn, XE1Yo, XE1Yp, XE2uz, XM0As, XM1Q3 |
| **Any cardiovascular disease** | 390-438, 440, 444, 445 | I00-I70, I730, I74- I75 |  |  |
| Hypertensive diseases |  | I10-I15 |  |  |
| Ischemic heart disease |  | I20-I25 |  |  |
| Embolism and thrombosis |  | I26, I74, I75 |  |  |
| Arrhythmia/conduction disorder |  | I44-I49 |  |  |
| Heart failure |  | I50 |  |  |
| Cerebrovascular disease |  | I60-I69 |  |  |
| Acute cardiovascular events |  | I21, I23, I24, I46, I60, I61, I63 |  |  |
| **Covariates: history of other psychiatric disorders** |  | F00-F31, F34-F39, F42, F44-F99 |  |  |

## **Table S2** Mapping between ICD-10 codes and Combined ICD codes for PheWAS analysis

| ICD-10 | Combined ICD codes | DESCRIPTION |
| --- | --- | --- |
| I00 | I00 | Acute rheumatic fever |
| I01 | I00 | Acute rheumatic fever |
| I02 | I00 | Acute rheumatic fever |
| I05 | I05 | Chronic rheumatic heart disease |
| I06 | I05 | Chronic rheumatic heart disease |
| I07 | I05 | Chronic rheumatic heart disease |
| I08 | I05 | Chronic rheumatic heart disease |
| I09 | I05 | Chronic rheumatic heart disease |
| I10 | I10 | Primary hypertension |
| I11 | I11 | Other hypertensive disorders |
| I12 | I11 | Other hypertensive disorders |
| I13 | I11 | Other hypertensive disorders |
| I15 | I11 | Other hypertensive disorders |
| I20 | I20 | Angina pectoris |
| I21 | I21 | Acute myocardial infarction |
| I22 | I22 | Other ischemic heart disease |
| I23 | I21 | Acute myocardial infarction |
| I24 | I21 | Acute myocardial infarction |
| I25 | I25 | Chronic ischemic heart disease |
| I26 | I26 | Embolism and thrombosis |
| I27 | I27 | Other pulmonary heart diseases |
| I28 | I27 | Other pulmonary heart diseases |
| I30 | I30 | Diseases of pericardium |
| I31 | I30 | Diseases of pericardium |
| I32 | I30 | Diseases of pericardium |
| I33 | I33 | Endocarditis |
| I34 | I34 | Non-rheumatic valve disorders |
| I35 | I34 | Non-rheumatic valve disorders |
| I36 | I34 | Non-rheumatic valve disorders |
| I37 | I34 | Non-rheumatic valve disorders |
| I38 | I33 | Endocarditis |
| I39 | I33 | Endocarditis |
| I40 | I40 | Myocarditis |
| I41 | I40 | Myocarditis |
| I42 | I42 | Cardiomyopathy |
| I43 | I42 | Cardiomyopathy |
| I44 | I49 | Other cardiac arrhythmias |
| I45 | I49 | Other cardiac arrhythmias |
| I46 | I46 | Cardiac arrest |
| I47 | I49 | Other cardiac arrhythmias |
| I48 | I48 | Atrial fibrillation and flutter |
| I49 | I49 | Other cardiac arrhythmias |
| I50 | I50 | Heart failure |
| I51 | I51 | Complications and ill-defined descriptions of heart disease |
| I52 | I52 | Other heart disorders in diseases classified elsewhere |
| I60 | I60 | Stroke |
| I61 | I60 | Stroke |
| I62 | I60 | Stroke |
| I63 | I60 | Stroke |
| I64 | I60 | Stroke |
| I65 | I65 | Other cerebrovascular diseases |
| I66 | I65 | Other cerebrovascular diseases |
| I67 | I65 | Other cerebrovascular diseases |
| I68 | I65 | Other cerebrovascular diseases |
| I69 | I69 | Sequelae of cerebrovascular disease |
| I70 | I70 | Atherosclerosis |
| I730 | I73 | Peripheral vascular disease |
| I74 | I26 | Embolism and thrombosis |
| I75 | I26 | Embolism and thrombosis |

## **Table S3** Information of GWAS summary statistics for six major subtypes of cardiovascular diseases

| **Subtype of cardiovascular diseases** | **PMID** | **Title** | **Odds ratio (95% confidence intervals)^1^** | **Nagelkerke’s R^2^** |  |
| --- | --- | --- | --- | --- | --- |
| Hypertensive diseases | 36653562 | FinnGen provides genetic insights from a well-phenotyped isolated population | 1.29 (1.28-1.31) | 0.88% |  |
| Ischemic heart disease | 33532862 | Genome-wide analysis identifies novel susceptibility loci for myocardial infarction | 1.56 (1.53-1.58) | 2.20% |  |
| Embolism and thrombosis | 36658437, 30305743, 36653562 | Genome-wide meta-analysis identifies 93 risk loci and enables risk prediction equivalent to monogenic forms of venous thromboembolism,  The UK Biobank resource with deep phenotyping and genomic data,  FinnGen provides genetic insights from a well-phenotyped isolated population | 1.37 (1.32-1.42) | 0.85% |  |
| Arrhythmia/conduction disorder | 30061737 | Biobank-driven genomic discovery yields new insight into atrial fibrillation biology | 1.55 (1.53-1.58) | 2.20% |  |
| Heart failure | 31919418 | Genome-wide association and Mendelian randomisation analysis provide insights into the pathogenesis of heart failure | 1.77 (1.72-1.83) | 2.73% |  |
| Cerebrovascular disease | 36180795 | Stroke genetics informs drug discovery and risk prediction across ancestries | 1.34 (1.31-1.38) | 0.83% |  |

**^1^** Odds ratio of the corresponding CVD for per standard deviation [SD] increase in PRS, derived from logistic regression model adjusted for birth year, sex, genotyping batch, and the first ten principal components for population heterogeneity.

**^2^** Nagelkerke’s R^2^ of the corresponding CVD for per standard deviation [SD] increase in PRS, derived from logistic regression model.

## **Table S4** Hazard ratios (HRs) with 95% confidence intervals (CIs) of cardiovascular disease among patients with common psychiatric disorders compared to matched unexposed individuals, by different psychiatric disorders

|  |  | **The whole study period** | | **≤ 6 months follow-up** | | **> 6 months follow-up** | |
| --- | --- | --- | --- | --- | --- | --- | --- |
| **Subtype of psychiatric disorders** | **Cardiovascular diseases** | **No. of cases (incidence^1^) in patients/matched individuals** | **HR (95% CIs) ^2^** | **No. of cases (incidence^1^) in patients/matched individuals** | **HR (95% CIs) ^2^** | **No. of cases (incidence^1^) in patients/matched individuals** | **HR (95% CIs) ^2^** |
| **Anxiety (n=11,740)** | **Any cardiovascular diseases** | 3553 (25.52)/14712 (21.28) | 1.18 (1.14-1.23) | 145 (25.28)/417 (14.5) | 1.77 (1.45-2.16) | 3408 (25.53)/14087 (21.47) | 1.17 (1.12-1.21) |
|  | Hypertensive diseases | 2740 (18.93)/11289 (15.85) | 1.17 (1.12-1.22) | 91 (15.82)/297 (10.32) | 1.54 (1.20-1.99) | 2649 (19.06)/10885 (16.03) | 1.16 (1.10-1.21) |
|  | Ischemic heart disease | 882 (5.65)/3355 (4.43) | 1.23 (1.13-1.33) | 36 (6.24)/92 (3.19) | 2.02 (1.31-3.11) | 846 (5.63)/3237 (4.46) | 1.21 (1.11-1.31) |
|  | Embolism and thrombosis | 150 (0.93)/644 (0.83) | 1.06 (0.88-1.28) | 6 (1.04)/7 (0.24) | 6.07 (1.59-23.13) | 144 (0.93)/636 (0.85) | 1.02 (0.84-1.24) |
|  | Arrhythmia/conduction disorder | 810 (5.12)/3389 (4.45) | 1.13 (1.04-1.22) | 15 (2.60)/54 (1.87) | 1.39 (0.78-2.48) | 795 (5.21)/3327 (4.55) | 1.12 (1.04-1.22) |
|  | Heart failure | 200 (1.24)/816 (1.05) | 1.03 (0.87-1.22) | 5 (0.87)/10 (0.35) | 2.67 (0.88-8.07) | 195 (1.25)/805 (1.08) | 1.01 (0.86-1.20) |
|  | Cerebrovascular disease | 326 (2.03)/1270 (1.65) | 1.19 (1.05-1.36) | 9 (1.56)/28 (0.97) | 1.62 (0.73-3.60) | 317 (2.05)/1240 (1.67) | 1.18 (1.04-1.35) |
|  | Acute cardiovascular events | 432 (2.71)/1771 (2.31) | 1.13 (1.01-1.26) | 13 (2.25)/43 (1.49) | 1.53 (0.82-2.88) | 419 (2.72)/1724 (2.34) | 1.12 (1.00-1.25) |
| **Depression (n=25,119)** | **Any cardiovascular diseases** | 8080 (25.63)/31386 (19.7) | 1.22 (1.19-1.26) | 292 (23.77)/757 (12.3) | 1.73 (1.51-2.00) | 7788 (25.7)/30128 (19.87) | 1.21 (1.18-1.24) |
|  | Hypertensive diseases | 6374 (19.42)/24122 (14.72) | 1.22 (1.19-1.26) | 193 (15.68)/511 (8.29) | 1.65 (1.38-1.96) | 6181 (19.57)/23335 (14.89) | 1.21 (1.18-1.25) |
|  | Ischemic heart disease | 2118 (5.93)/7212 (4.15) | 1.31 (1.24-1.37) | 67 (5.43)/167 (2.71) | 1.72 (1.27-2.33) | 2051 (5.95)/6999 (4.19) | 1.29 (1.23-1.36) |
|  | Embolism and thrombosis | 416 (1.13)/1469 (0.83) | 1.23 (1.10-1.38) | 7 (0.57)/20 (0.32) | 1.81 (0.70-4.68) | 409 (1.14)/1449 (0.85) | 1.22 (1.09-1.37) |
|  | Arrhythmia/conduction disorder | 1686 (4.64)/6912 (3.95) | 1.10 (1.04-1.16) | 29 (2.35)/107 (1.73) | 1.29 (0.85-1.97) | 1657 (4.72)/6798 (4.03) | 1.10 (1.04-1.16) |
|  | Heart failure | 518 (1.40)/1716 (0.97) | 1.26 (1.13-1.39) | 7 (0.57)/13 (0.21) | 2.67 (1.01-7.07) | 511 (1.43)/1702 (0.99) | 1.25 (1.12-1.39) |
|  | Cerebrovascular disease | 809 (2.20)/2582 (1.46) | 1.40 (1.29-1.52) | 26 (2.11)/36 (0.58) | 3.62 (2.05-6.40) | 783 (2.20)/2544 (1.49) | 1.38 (1.27-1.50) |
|  | Acute cardiovascular events | 1022 (2.79)/3781 (2.15) | 1.17 (1.09-1.26) | 32 (2.59)/73 (1.18) | 1.86 (1.18-2.93) | 990 (2.80)/3701 (2.18) | 1.16 (1.08-1.25) |
| **Stress-related disorder (n=7,646)** | **Any cardiovascular diseases** | 2163 (24.06)/9049 (20.32) | 1.13 (1.07-1.19) | 80 (21.38)/237 (12.65) | 1.61 (1.23-2.11) | 2083 (24.18)/8704 (20.57) | 1.12 (1.06-1.17) |
|  | Hypertensive diseases | 1690 (18.19)/7068 (15.44) | 1.12 (1.06-1.18) | 55 (14.68)/157 (8.37) | 1.80 (1.29-2.52) | 1635 (18.34)/6848 (15.70) | 1.10 (1.04-1.17) |
|  | Ischemic heart disease | 520 (5.18)/2023 (4.17) | 1.18 (1.07-1.31) | 13 (3.46)/47 (2.50) | 1.47 (0.78-2.77) | 507 (5.24)/1970 (4.23) | 1.18 (1.07-1.31) |
|  | Embolism and thrombosis | 82 (0.79)/399 (0.80) | 0.94 (0.73-1.21) | 3 (0.80)/11 (0.59) | 1.40 (0.37-5.28) | 79 (0.79)/387 (0.81) | 0.93 (0.72-1.20) |
|  | Arrhythmia/conduction disorder | 495 (4.88)/1992 (4.07) | 1.14 (1.03-1.27) | 13 (3.46)/32 (1.70) | 2.28 (1.15-4.49) | 482 (4.93)/1956 (4.16) | 1.13 (1.02-1.25) |
|  | Heart failure | 128 (1.24)/523 (1.05) | 1.08 (0.88-1.34) | 3 (0.80)/5 (0.27) | 1.22 (0.21-7.15) | 125 (1.26)/518 (1.09) | 1.08 (0.88-1.33) |
|  | Cerebrovascular disease | 189 (1.84)/709 (1.43) | 1.21 (1.02-1.43) | 9 (2.39)/18 (0.96) | 2.55 (1.07-6.10) | 180 (1.82)/690 (1.45) | 1.18 (1.00-1.41) |
|  | Acute cardiovascular events | 262 (2.56)/1042 (2.11) | 1.12 (0.97-1.29) | 11 (2.92)/30 (1.60) | 1.74 (0.85-3.58) | 251 (2.55)/1008 (2.13) | 1.11 (0.96-1.28) |

^1^ Per 1000 person years.

^2^ Hazard ratios (with 95% confidence intervals) of any or subtypes of cardiovascular diseases in individual with common psychiatric disorders compared to sex and year of birth matched unexposed individuals, derived from Cox regression models and adjusted for educational level, ethnicity, smoking status, BMI, history of other psychiatric disorders, family history of CVD, and CCI score. For subtypes of cardiovascular diseases with less than 20 cases in patients with common psychiatric disorders or matched unexposed individuals, hazard rations were derived from Cox regression models and adjusted for educational level and ethnicity. Definition of subtypes cardiovascular diseases by ICD-10 codes can be found in the Table S1. Time since index date was used as time scale.

**Table S5** Hazard ratios (HRs) with 95% confidence intervals (CIs) of cardiovascular disease among patients with common psychiatric disorders compared to matched unexposed individuals, by different diagnosis source of common psychiatric disorders

|  | **The whole study period** | | **≤ 6 months follow-up** | | **> 6 months follow-up** | |
| --- | --- | --- | --- | --- | --- | --- |
| **Diagnosis source of common psychiatric disorders** | **No. of cases (incidence^1^) in patients/matched individuals** | **HR (95% CI)^2^** | **No. of cases (incidence^1^) in patients/matched individuals** | **HR (95% CI) ^2^** | **No. of cases (incidence^1^) in patients/matched individuals** | **HR (95% CI) ^2^** |
| Only self-reported or primary care data | 13014 (24.83)/52777 (20.16) | 1.17 (1.15-1.20) | 471 (22.57)/1344 (12.86) | 1.63 (1.46-1.82) | 12543 (24.92)/50679 (20.35) | 1.16 (1.14-1.19) |
| Inpatient hospital data | 782 (38.75)/2370 (21.23) | 1.81 (1.65-1.98) | 46 (51.52)/67 (14.81) | 3.39 (2.26-5.08) | 736 (38.16)/2240 (21.32) | 1.75 (1.59-1.92) |
| *p for difference^3^* |  | <0.0001 |  | 0.0005 |  | <0.0001 |

^1^ Per 1000 person years.

^2^ Cox regression models were stratified by diagnosis source of common psychiatric disorders, and adjusted for sex, birth year, educational level, ethnicity, smoking status, TDI, BMI, history of other psychiatric disorders, and CCI score.

^3^ The differences in hazard ratios for different age of psychiatric disorders diagnosis were assessed between different diagnosis source of common psychiatric disorders by Wald test.

**Table S6** Hazard ratios (HRs) with 95% confidence intervals (CIs) of cardiovascular disease among patients with common psychiatric disorders compared to matched unexposed individuals, by different age of psychiatric disorders diagnosis

|  | **The whole study period** | | **≤ 6 months follow-up** | | **> 6 months follow-up** | |
| --- | --- | --- | --- | --- | --- | --- |
| **Age of psychiatric disorders diagnosis (thirds), years** | **No. of cases (incidence^1^) in patients/matched individuals** | **HR (95% CI)^2^** | **No. of cases (incidence^1^) in patients/matched individuals** | **HR (95% CI) ^2^** | **No. of cases (incidence^1^) in patients/matched individuals** | **HR (95% CI) ^2^** |
| <47 | 3000 (14.06)/9689 (9.91) | 1.25 (1.20-1.31) | 63 (9.139)/118 (3.682) | 2.25 (1.60-3.17) | 2937 (14.22)/9519 (10.11) | 1.24 (1.19-1.30) |
| 47-55 | 4259 (24.19)/14492 (19.13) | 1.18 (1.13-1.22) | 128 (18.33)/287 (9.578) | 1.70 (1.36-2.13) | 4131 (24.43)/14058 (19.49) | 1.17 (1.12-1.21) |
| >55 | 6537 (42.21)/25966 (35.05) | 1.18 (1.15-1.22) | 326 (41.33)/909 (24.53) | 1.66 (1.45-1.89) | 6211 (42.26)/24482 (35.45) | 1.16 (1.13-1.20) |
| *p for difference^3^* |  | 0.0327 |  | 0.1040 |  | 0.0145 |

^1^ Per 1000 person years.

^2^ Cox regression models were stratified by diagnosis source of common psychiatric disorders, and adjusted for sex, birth year, educational level, ethnicity, smoking status, TDI, BMI, history of other psychiatric disorders, and CCI score.

^3^ The differences in hazard ratios for different age of psychiatric disorders diagnosis were assessed between age of <47 and age of >55 subgroups by Wald test.

## **Table S7** Odds ratios (ORs) with 95% confidence intervals (CIs) for the significant pairs of cardiovascular diseases following a diagnosis of common psychiatric disorders

| **Codes for CVD pairs^1^** | **CVD 1** | **CVD 2** | **Case numbers^2^** | **OR (95% CIs)^3^** |
| --- | --- | --- | --- | --- |
| I10→I20 | Primary hypertension | Angina pectoris | 789 | 5.24 (4.46-6.17) |
| I21→I20 | Acute myocardial infarction | Angina pectoris | 243 | 20.93 (13.23-33.11) |
| I25→I20 | Chronic ischemic heart disease | Angina pectoris | 644 | 20.48 (15.41-27.22) |
| I10→I21 | Primary hypertension | Acute myocardial infarction | 375 | 4.32 (3.44-5.41) |
| I10→I25 | Primary hypertension | Chronic ischemic heart disease | 1137 | 4.44 (3.91-5.05) |
| I21→I25 | Acute myocardial infarction | Chronic ischemic heart disease | 281 | 49.08 (26.08-92.35) |
| I10→I49 | Primary hypertension | Other cardiac arrhythmias | 711 | 3.28 (2.78-3.86) |
| I20→I49 | Angina pectoris | Other cardiac arrhythmias | 201 | 3.17 (2.46-4.09) |
| I25→I49 | Chronic ischemic heart disease | Other cardiac arrhythmias | 310 | 4.58 (3.63-5.79) |
| I21→I50 | Acute myocardial infarction | Heart failure | 104 | 7.48 (4.67-11.96) |
| I10→I50 | Primary hypertension | Heart failure | 588 | 7.27 (5.73-9.23) |
| I20→I50 | Angina pectoris | Heart failure | 200 | 6.44 (4.65-8.93) |
| I25→I50 | Chronic ischemic heart disease | Heart failure | 281 | 6.21 (4.69-8.22) |
| I20→I51 | Angina pectoris | Complications and ill-defined descriptions of heart disease | 193 | 4.82 (3.54-6.57) |
| I25→I51 | Chronic ischemic heart disease | Complications and ill-defined descriptions of heart disease | 274 | 5.85 (4.44-7.71) |
| I10→I51 | Primary hypertension | Complications and ill-defined descriptions of heart disease | 540 | 5.52 (4.37-6.97) |
| I10→I60 | Primary hypertension | Stroke | 338 | 3.12 (2.47-3.95) |
| I20→I65 | Angina pectoris | Other cerebrovascular diseases | 121 | 3.52 (2.44-5.09) |
| I25→I65 | Chronic ischemic heart disease | Other cerebrovascular diseases | 142 | 3.09 (2.23-4.29) |
| I10→I65 | Primary hypertension | Other cerebrovascular diseases | 416 | 4.58 (3.55-5.91) |
| I60→I65 | Stroke | Other cerebrovascular diseases | 109 | 14.87 (7.93-27.90) |
| I10→I69 | Primary hypertension | Sequelae of cerebrovascular disease | 147 | 7.84 (4.88-12.61) |
| I60→I69 | Stroke | Sequelae of cerebrovascular disease | 145 | 74.38 (23.66-233.86) |

A total of 23 CVD pairs with N ≥ 100 and statistically significant odds ratios after Bonferroni correction for patients with common psychiatric disorders were shown in this table;

Abbreviation: CVD, cardiovascular disease;

^1^Combined ICD-10 code for two CVDs; mapping between the original ICD-10 code and the combined ICD-10 code can be found in the Table S2;

^2^Number of patients with common psychiatric disorders who experienced the corresponding pairs of two CVDs;

^3^Odds ratios (with 95% confidence intervals) of pairs of two CVDs;

^4^Number of individuals with psychiatric disorders having the same diagnosis date of two CVDs divided by the number of patients with common psychiatric disorders diagnosed with both two CVDs.

## **Table S8** Hazard ratios (HRs) with 95% confidence intervals (CIs) of primary diagnosis of cardiovascular disease among patients with common psychiatric disorders compared to matched unexposed individuals

|  | **The whole study period** | | **≤ 6 months follow-up** | | **> 6 months follow-up** | |
| --- | --- | --- | --- | --- | --- | --- |
|  | **No. of cases (incidence^1^) in patients/matched individuals** | **HR (95% CI) ^2^** | **No. of cases (incidence^1^) in patients/matched individuals** | **HR (95% CI) ^2^** | **No. of cases (incidence^1^) in patients/matched individuals** | **HR (95% CI) ^2^** |
| **Model information** |  |  |  |  |  |  |
| Controlled for sex, year of birth, TDI, ethnicity and educational level | 2433(4.47)/10034(3.68) | 1.25 (1.19-1.30) | 124(5.70)/306(2.81) | 2.00 (1.62-2.46) | 2309(4.42)/9571(3.69) | 1.22 (1.16-1.28) |
| As above + BMI+ smoking status+ history of other psychiatry disorders+ CCI |  | 1.18 (1.13-1.24) |  | 1.92 (1.55-2.39) |  | 1.16 (1.10-1.21) |
| As above + family history of CVDs |  | 1.17 (1.12-1.23) |  | 1.94 (1.56-2.41) |  | 1.15 (1.10-1.21) |
| **Subtypes of cardiovascular diseases^3^** |  |  |  |  |  |  |
| Hypertensive diseases | 171 (0.27)/700 (0.23) | 1.11 (0.93-1.32) | 5 (0.23)/13 (0.12) | 1.90 (0.68-5.33) | 166 (0.27)/686 (0.23) | 1.10 (0.92-1.31) |
| Ischemic heart disease | 2338 (3.8)/8593 (2.9) | 1.23 (1.17-1.29) | 80 (3.7)/212 (1.9) | 1.75 (1.33-2.31) | 2258 (3.8)/8345 (2.9) | 1.22 (1.16-1.28) |
| Embolism and thrombosis | 457 (0.72)/1667 (0.55) | 1.21 (1.08-1.35) | 12 (0.55)/26 (0.24) | 2.30 (1.16-4.57) | 445 (0.72)/1639 (0.56) | 1.19 (1.07-1.33) |
| Arrhythmia/conduction disorder | 1203 (1.9)/5347 (1.8) | 1.04 (0.97-1.11) | 31 (1.4)/104 (0.95) | 1.45 (0.94-2.23) | 1172 (1.9)/5232 (1.8) | 1.03 (0.97-1.10) |
| Heart failure | 234 (0.37)/783 (0.26) | 1.23 (1.05-1.44) | 5 (0.23)/6 (0.055) | 5.42 (1.40-20.89) | 229 (0.37)/776 (0.26) | 1.22 (1.04-1.43) |
| Cerebrovascular disease | 832 (1.3)/2938 (0.96) | 1.26 (1.17-1.37) | 31 (1.4)/49 (0.45) | 2.78 (1.69-4.56) | 801 (1.3)/2879 (0.98) | 1.24 (1.14-1.34) |
| Acute cardiovascular events | 1478 (2.3)/5564 (1.8) | 1.17 (1.10-1.24) | 50 (2.3)/95 (0.87) | 2.23 (1.53-3.26) | 1428 (2.3)/5446 (1.9) | 1.15 (1.08-1.22) |

^1^Per 1000 person years.

^2^Hazard ratios (with 95% confidence intervals) of cardiovascular disease in patients with common psychiatric disorders compared to matched unexposed individuals, derived from Cox regression models, stratified by matching identifiers and adjusted for covariates listed in model information column.

^3^Hazard ratios (with 95% confidence intervals) of subtypes of cardiovascular diseases in individual with psychiatric disorders compared to matched unexposed individuals, derived from Cox regression models, stratified by matching identifiers and adjusted for sex, birth year, educational level, ethnicity, smoking status, TDI, BMI, history of other psychiatric disorders, family history of CVD, and CCI score. For subtypes of cardiovascular diseases with less than 20 cases in patients with common psychiatric disorders, hazard rations were derived from Cox regression models, stratified by matching identifiers and adjusted for sex, birth year, and TDI. Definition of subtypes cardiovascular diseases by ICD-10 codes can be found in the Table S1.

## **Table S9** Hazard ratios (HRs) with 95% confidence intervals (CIs) of primary diagnosis of cardiovascular disease among patients with common psychiatric disorders compared to matched unexposed individuals, by different disease susceptibility

|  | **≤ 6 months follow-up** | | **> 6 months follow-up** | |
| --- | --- | --- | --- | --- |
| **Disease susceptibility** | **No. of cases (incidence^1^) in patients/matched individuals** | **HR (95% CI)^2^** | **No. of cases (incidence^1^) in patients/matched individuals** | **HR (95% CI)^2^** |
| **Family history of cardiovascular disease** | | | | |
| No | 42 (4.57)/41 (1.91) | 3.04 (1.77-5.23) | 802 (3.56)/1424 (2.74) | 1.16 (1.05-1.28) |
| Yes | 82 (6.52)/122 (3.42) | 1.98 (1.46-2.69) | 1507 (5.07)/3652 (4.35) | 1.15 (1.08-1.23) |
| *p for difference^3^* |  | 0.1767 |  | 0.8861 |
| **Level of polygenic risk score (PRS) of the corresponding cardiovascular disease^4^** | | | | |
| **Hypertensive diseases** |  |  |  |  |
| Low PRS (<the lower tertile) | 2 (0.34)/1 (0.13) | NA | 28 (0.17)/39 (0.18) | 0.69 (0.36-1.36) |
| Moderate PRS (lower-upper tertiles) | 2 (0.32)/0 (0) | NA | 42 (0.24)/52 (0.23) | 0.88 (0.53-1.46) |
| High PRS (>the upper tertile) | 1 (0.16)/1 (0.12) | NA | 57 (0.32)/59 (0.26) | 1.16 (0.75-1.80) |
| *p for difference^3^* |  | NA |  | 0.2009 |
| **Ischemic heart disease** |  |  |  |  |
| Low PRS (<the lower tertile) | 11 (1.88)/2 (0.25) | 11.16 (1.12-111.18) | 334 (2.06)/338 (1.56) | 1.24 (1.04-1.49) |
| Moderate PRS (lower-upper tertiles) | 11 (1.78)/10 (1.2) | 2.38 (1.27-4.45) | 512 (2.99)/553 (2.5) | 1.17 (1.01-1.35) |
| High PRS (>the upper tertile) | 48 (7.7)/29 (3.48) | 2.22 (0.24-20.49) | 1118 (6.62)/1149 (5.24) | 1.17 (1.06-1.28) |
| *p for difference^3^* |  | 0.3224 |  | 0.5748 |
| **Embolism and thrombosis** |  |  |  |  |
| Low PRS (<the lower tertile) | 2 (0.33)/3 (0.36) | NA | 86 (0.5)/84 (0.37) | 1.16 (0.78-1.70) |
| Moderate PRS (lower-upper tertiles) | 5 (0.81)/3 (0.36) | 2.73 (0.48-15.56) | 119 (0.69)/127 (0.57) | 1.06 (0.78-1.45) |
| High PRS (>the upper tertile) | 5 (0.83)/4 (0.49) | NA | 191 (1.12)/174 (0.79) | 1.50 (1.19-1.90) |
| *p for difference^3^* |  | NA |  | 0.2679 |
| **Arrhythmia/conduction disorder** |  |  |  |  |
| Low PRS (<the lower tertile) | 4 (0.66)/4 (0.48) | 3.69 (0.51-26.64) | 160 (0.94)/246 (1.09) | 0.82 (0.64-1.04) |
| Moderate PRS (lower-upper tertiles) | 7 (1.16)/7 (0.85) | 12.11 (0.70-209.43) | 236 (1.4)/321 (1.46) | 0.95 (0.78-1.16) |
| High PRS (>the upper tertile) | 18 (2.9)/16 (1.89) | 2.45 (1.05-5.73) | 664 (3.87)/753 (3.34) | 1.15 (1.01-1.30) |
| *p for difference^3^* |  | 0.7092 |  | 0.0154 |
| **Heart failure** |  |  |  |  |
| Low PRS (<the lower tertile) | 0 (0)/1 (0.12) | NA | 30 (1.8)/34 (0.15) | 1.50 (0.81-2.77) |
| Moderate PRS (lower-upper tertiles) | 0 (0)/0 (0) | NA | 35 (0.2)/39 (0.17) | 0.62 (0.30-1.24) |
| High PRS (>the upper tertile) | 5 (0.8)/3 (0.36) | 2.22 (0.24-20.49) | 134 (0.76)/119 (0.52) | 1.28 (0.94-1.75) |
| *p for difference^3^* |  | NA |  | 0.6518 |
| **Cerebrovascular disease** |  |  |  |  |
| Low PRS (<the lower tertile) | 4 (0.66)/5 (0.61) | 1.20 (0.12-11.94) | 156 (0.92)/179 (0.81) | 1.05 (0.81-1.36) |
| Moderate PRS (lower-upper tertiles) | 7 (1.15)/2 (0.24) | 10.45 (0.55-197.38) | 207 (1.21)/188 (0.84) | 1.41 (1.11-1.79) |
| High PRS (>the upper tertile) | 14 (2.26)/8 (0.95) | 2.48 (0.80-7.64) | 363 (2.11)/407 (1.8) | 1.06 (0.90-1.26) |
| *p for difference^3^* |  | 0.5451 |  | 0.5789 |

^1^ Per 1000 person years.

^2^ Cox regression models were stratified by matching identifiers and adjusted for sex, TDI, year of birth, educational level, ethnicity, smoking status, BMI, history of other psychiatry disorders, and CCI score.

^3^ The differences in hazard ratios for family history of CVD were assessed by Wald test. The differences in hazard ratios for CVD PRS were assessed between low and high subgroups by Wald test.

^4^ Polygenic risk scores of the corresponding cardiovascular diseases were calculated based on published GWAS summary statistics data.

## **Table S10** Odds ratios (ORs) with 95% confidence intervals (CIs) for the significant pairs of primary diagnosis of cardiovascular diseases following a diagnosis of common psychiatric disorders

| **Codes for CVD pairs^1^** | **CVD 1** | **CVD 2** | **Case numbers^2^** | **OR (95% CIs)^3^** |
| --- | --- | --- | --- | --- |
| I21→I20 | Acute myocardial infarction | Angina pectoris | 118 | 16.19 (9.10-28.80) |
| I21→I25 | Acute myocardial infarction | Chronic ischemic heart disease | 264 | 47.42 (25.19-89.26) |
| I25→I60 | Chronic ischemic heart disease | Stroke | 63 | 2.73 (1.79-4.17) |
| I60→I65 | Stroke | Other cerebrovascular diseases | 29 | 16.67 (5.03-55.20) |

A total of 4 CVDs pairs with N ≥ 20 and statistically significant odds ratios after Bonferroni correction for individuals with psychiatric disorders were show in this table;

Abbreviation: CVD, cardiovascular disease;

^1^Combined ICD-10 code for two CVDs; mapping between the original ICD-10 code and the combined ICD-10 code can be found in the Table S2;

^2^Number of patients with common psychiatric disorders who experienced the corresponding pairs of two CVDs;

^3^Odds ratios (with 95% confidence intervals) of pairs of two CVDs;

^4^Number of individuals with psychiatric disorders having the same diagnosis date of two CVDs divided by the number of patients with common psychiatric disorders diagnosed with both two CVDs.


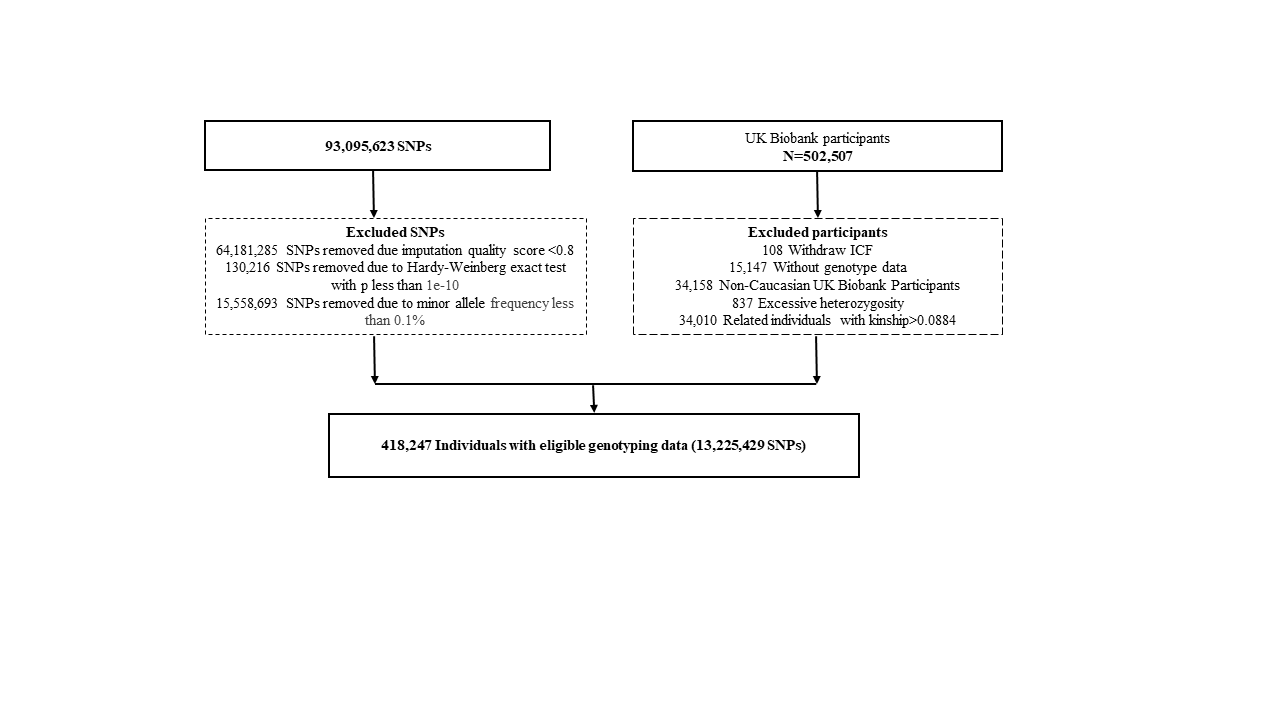


**Fig. S1** Flowchart of selection on participants and SNPs for PRS calculation


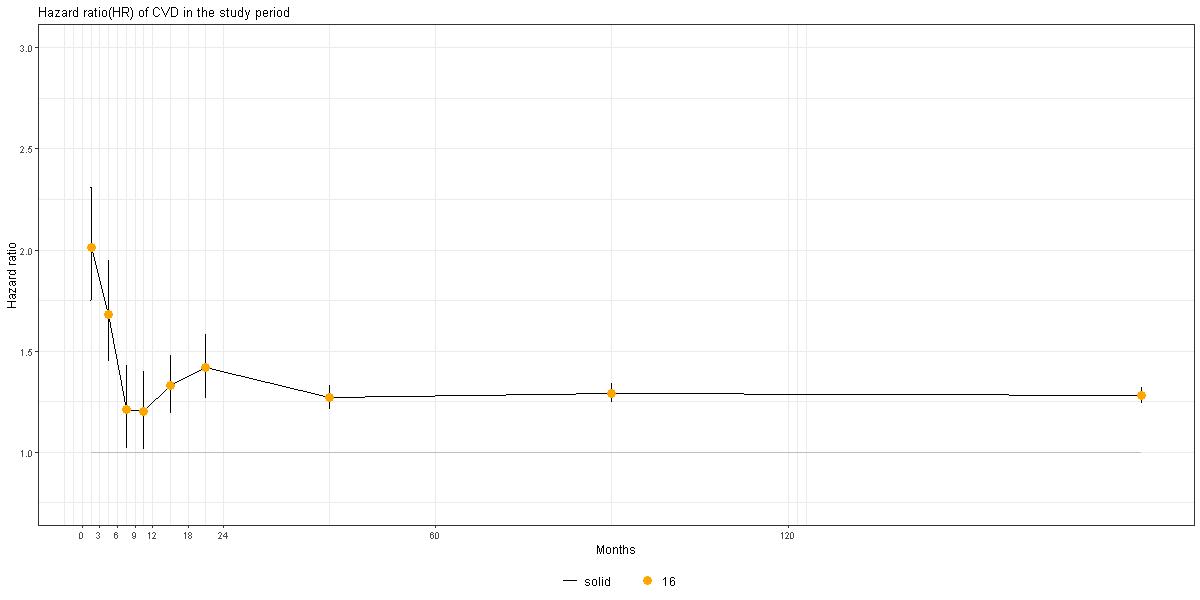


## **Fig. S2** Hazard ratios (95% CIs) of cardiovascular disease among patients with common psychiatric disorders compared with their matched unexposed individuals, stratified by time of follow-up

## Abbreviation: CI: confidence interval.

The X axis shows the different follow-up period: ≤3, 3-6, 6-12, 12-18, 18-24, 24-60, 60-120, 120-240, and >240 months follow-up. The Y axis shows the significant hazard ratios of cardiovascular disease among patients with common psychiatric disorders compared with their matched unexposed individuals, derived from Cox models adjusted for sex, birth year and ethnicity.


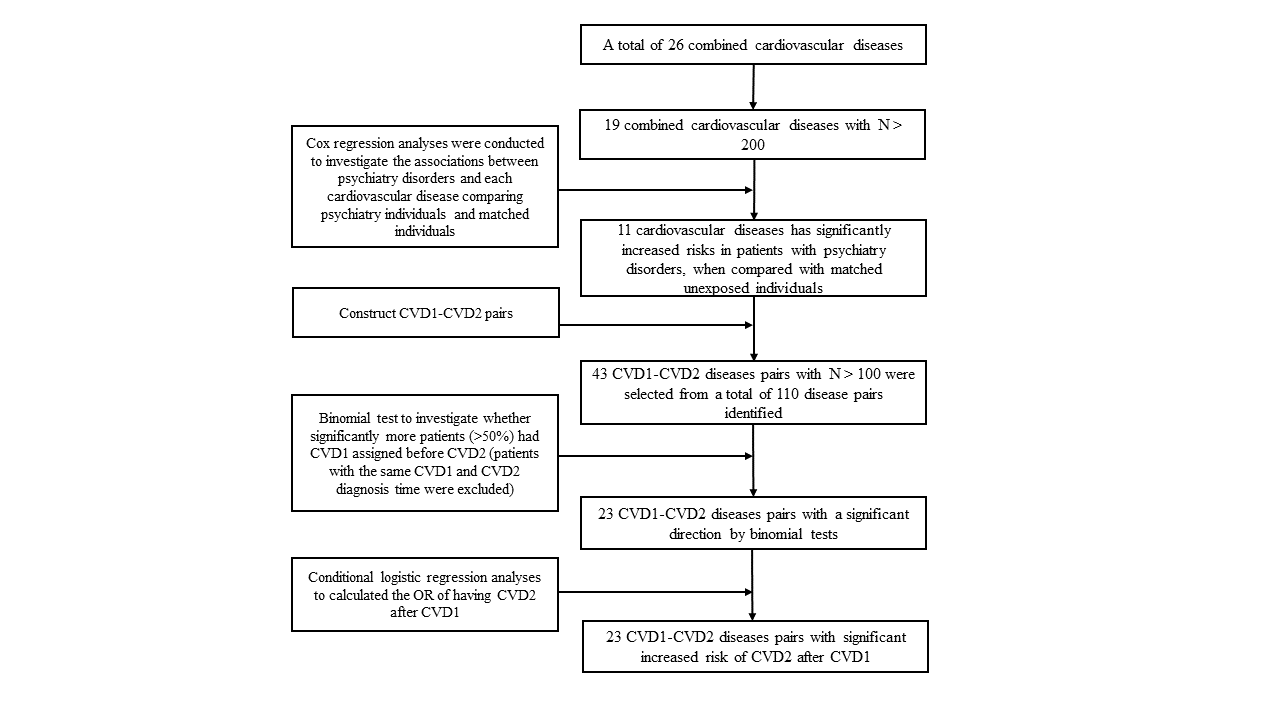


**Fig. S3** Flow chart of identifying trajectory progression of cardiovascular disease following a diagnosis of common psychiatric disorders


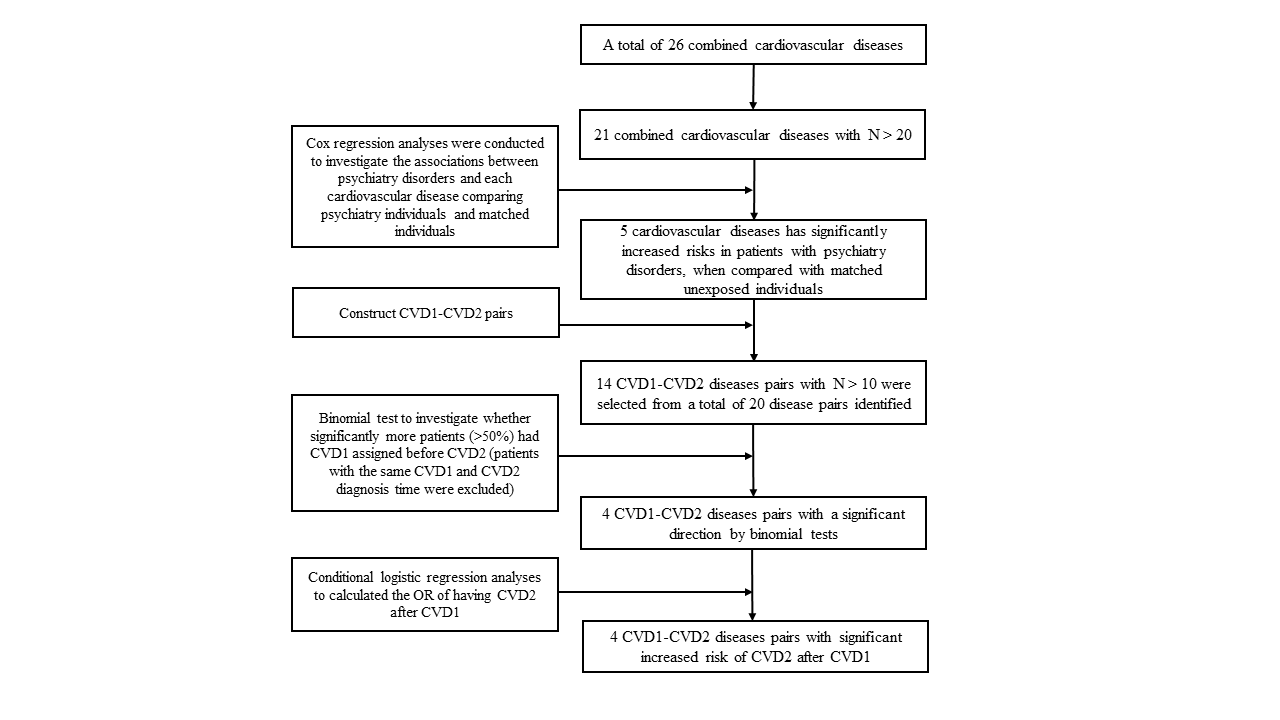


**Fig. S4** Flow chart of identifying trajectory progression of primary diagnosis of cardiovascular disease following a diagnosis of common psychiatric disorders


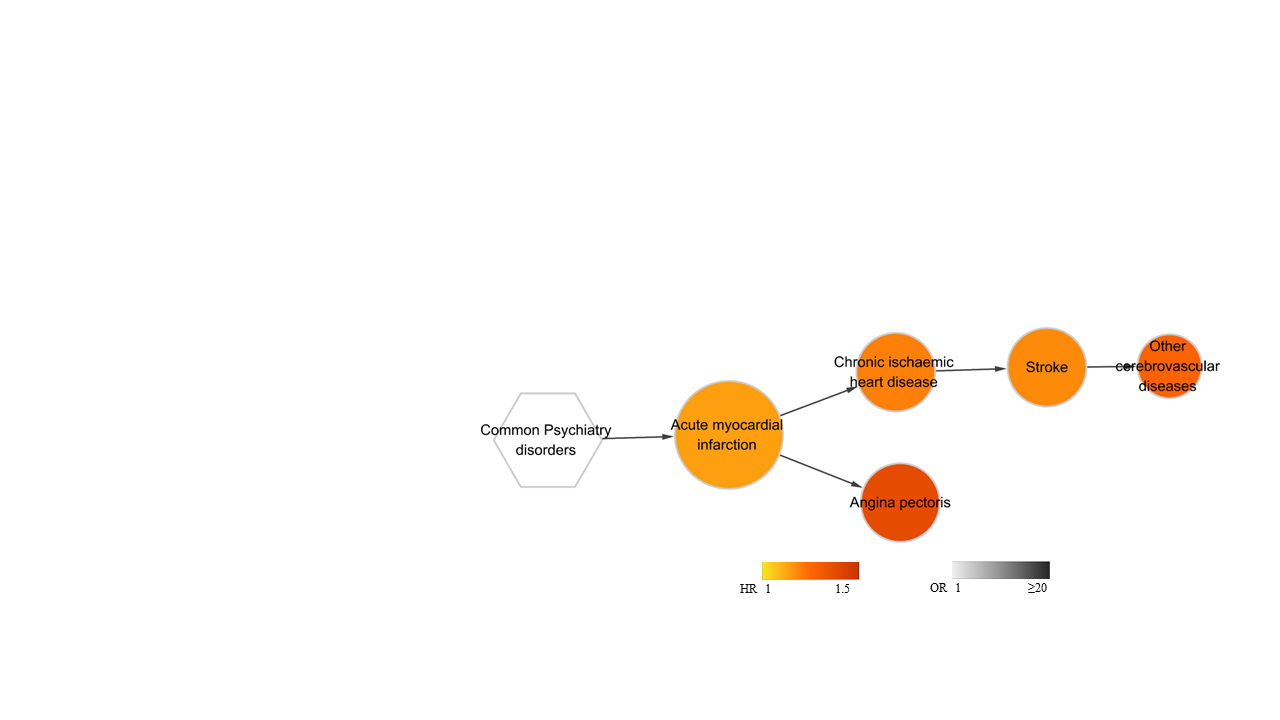


**Fig. S5** Trajectory progression of primary diagnosis of cardiovascular disease following a diagnosis of common psychiatric disorders

This figure illustrates following trajectory progression of primary diagnosis of cardiovascular disease identified in our analysis. The combined cardiovascular diseases are shown within the circle. The color of the circle represents the hazard ratios of this cardiovascular disease when comparing patients with common psychiatric disorders to matched unexposed individuals. The color of the arrows indicates the odds ratio of the sequential association between the two cardiovascular disease events among patients with common psychiatric disorders.
